# Riboflavin and pantothenic acid biosynthesis are crucial for iron homeostasis and virulence in the pathogenic mold *Aspergillus fumigatus*

**DOI:** 10.1080/21505594.2018.1482181

**Published:** 2018-07-27

**Authors:** Anna-Maria Dietl, Zohar Meir, Yona Shadkchan, Nir Osherov, Hubertus Haas

**Affiliations:** a Division of Molecular Biology, Biocenter, Medical University of Innsbruck, Innsbruck, Austria; b Department of Clinical Microbiology and Immunology, Sackler School of Medicine Ramat-Aviv, Tel-Aviv, Israel

**Keywords:** *Aspergillus fumigatus* virulence, vitamin B biosynthesis, auxotrophy, iron metabolism, antifungal drug target

## Abstract

**Background**: *Aspergillus fumigatus* is the most prevalent airborne fungal pathogen, causing invasive fungal infections mainly in immunosuppressed individuals. Death rates from invasive aspergillosis remain high because of limited treatment options and increasing antifungal resistance. The aim of this study was to identify key fungal-specific genes participating in vitamin B biosynthesis in A. *fumigatus*. Because these genes are absent in humans they can serve as possible novel targets for antifungal drug development. **Methods**: By sequence homology we identified, deleted and analysed four key A. *fumigatus genes (riboB, panA, pyroA, thiB)* involved respectively in the biosynthesis of riboflavin (vitamin B2), pantothenic acid (vitamin B5), pyridoxine (vitamin B6) and thiamine (vitamin B1). **Results**: Deletion of riboB, panA, pyroA or thiB resulted in respective vitamin auxotrophy. Lack of riboflavin and pantothenic acid biosynthesis perturbed many cellular processes including iron homeostasis. Virulence in murine pulmonary and systemic models of infection was severely attenuated following deletion of *riboB* and *panA*, strongly reduced after *pyroA* deletion and weakly attenuated after *thiB* deletion. **Conclusions**: This study reveals the biosynthetic pathways of the vitamins riboflavin and pantothenic acid as attractive targets for novel antifungal therapy. Moreover, the virulence studies with auxotrophic mutants serve to identify the availability of nutrients to pathogens in host niches.

**Abbreviations**: BPS: bathophenanthrolinedisulfonate; BSA: bovine serum albumin; CFU: colony forming unit; -Fe: iron starvation; +Fe: iron sufficiency; hFe: high iron; NRPSs: nonribosomal peptide synthetases; PKSs: polyketide synthaseses; wt: wild type

## Introduction

Invasive fungal infections have increased dramatically in number over the last 40 years, largely because of the increase in the global number of immunocompromised patients. It is estimated that over 1.5 million people/year are infected worldwide by the major invasive fungal pathogens *Candida albicans, Aspergillus fumigatus* and *Cryptococcus neoformans* [1,2]. Despite modern antifungal treatments, mortality rates remain between 20 to 90%. Treatment efficacy has plateaued and fungal resistance is developing [3].

Development of new drugs is hampered by the fact that fungi are eukaryotes and share with humans many of the same metabolic pathways. Therefore, most existing antifungals are not sufficiently selective and have various side effects.

Research has therefore focused on identifying essential genes and pathways not shared with the infected host [4–7]. Using a combination of comparative genomics, transcriptomics and metabolic flux analysis, we recently identified 64 fungal-specific targets including metabolic enzymes participating in amino acid, lipid and vitamin biosynthesis [8]. Of these, 18 targets have already been validated in the literature, including enzymes participating in the biosynthesis of aromatic amino acids [9], lysine [10,11], histidine [12] and cysteine/methionine [13] and in the biosynthesis of lipids, including phospholipids [14], fatty acids [6] and oxylipins [15]. However, the assessment of fungal-specific targets in the vitamin biosynthesis pathways and their importance during infection has not been well established.

In this study, we analysed for the first time the vitamin B biosynthetic pathways for thiamine (B1), riboflavin (B2), pantothenic acid (B5) and pyridoxine (B6) in the important human pathogenic mold *A. fumigatus*. These fungal pathways, including the encoding genes are shown in Supplementary Figure 1. They are not found in humans, who need to obtain the required vitamin intake from their food. We deleted key fungal-specific genes in each pathway and phenotypically evaluated the resulting auxotrophic mutants *in vitro* and *in vivo* during infection. We show that riboflavin and pantothenic acid biosynthesis are important for iron homeostasis *in vitro* and for establishing a lethal infection in mice, suggesting strategies for their development as antifungal targets.

## Materials and methods

### Strains and growth conditions

Fungal strains were generally cultured on/in *Aspergillus* minimal medium (AMM) containing 1% glucose as carbon source and 20 mM glutamine as nitrogen source or on complete medium containing 2 g/L peptone and 1 g/L yeast extract [16]. Alternatively, YAG medium consisting of 0.5% yeast extract, 1% glucose, 10 mM MgCl_2_, trace elements and vitamins, served as nutrient source. Supplements are indicated in the respective experiments. Iron was omitted for iron-depleted conditions (-Fe) and 30 µM FeSO_4_ was added into the media for iron-replete (+Fe) conditions. Vitamin stock solution includes 2.5 µM pantothenic acid, 10 µM pyridoxine and 10 µM niacin. Blood agar medium contained 1.8% agar, 0.5% sodium chloride and 10% or 25% blood. A hypoxic chamber (C-Chamber and Pro-Ox, Pro-CO_2_ controller; Biosherics) with the settings 1% O_2_, 5% CO_2_ and 94% N_2_ was used for hypoxic conditions. For growth assays, 10^4^ conidia were point-inoculated on minimal medium agar plates and incubated for 48 h at 37°C. For inoculation of 100 ml liquid media, 10^6^/ml conidia were used. The *akuA*-deficient derivative of ATCC46645 AfS77, termed wild-type (wt) here, served as the reference recipient [17]. Primers used in this study are listed in Supplementary Table 1. Generation of the *A. fumigatus* mutant strains and their verification by PCR and Southern analysis is outlined in detail in Supplementary Figure 2A-M. Strains used in this study are listed in Supplementary Table 2.

**Table 1.** **Flavoprotein-dependent pathways in *A. fumigatus***. The number of proteins are given within brackets. The complete list of identified flavoproteins (238 proteins) is found in supplementary table 7.

Pathway (number of proteins)
Metalloreduction, e.g. reductive iron assimilation [17]
Light regulation/response [4]
Ergosterol biosynthesis [6]
Amino acid biosynthesis [3]
Respiration/TCA cycle [1]
ß-Oxidation [4]
Purine catabolism [1]
Nitrate assimilation/NO detoxification [5]
REDOX homeostasis [4]
Pyridoxin biosynthesis [1]
Pantothenic acid biosynthesis [4]
Nicotinic acid biosynthesis [2]
Coenzyme A biosynthesis [2]
Siderophore biosynthesis [1]
Sulfur assimilation/methionine biosynthesis [4]
Iron-sulfur-cluster biosynthesis [2]
Secondary metabolism [13]
Ubiquinone biosynthesis [2]
Glycerophospholipid metabolism [1]
tRNA modification [7]
Autophagy [1]
Protein modification: sumoylation, neddylation, ubiquitination [6]

### Analysis of extra-and intracellular siderophores and biomass production

For quantification of extra- or intracellular siderophores, culture supernatants or lyophilized mycelia were saturated with FeSO_4_ and extracted as described previously [18]. To analyse biomass production, mycelia from liquid cultures were freeze-dried and weighed.

For the vitamin-shift experiment, fungal strains were inoculated in liquid minimal medium supplemented with vitamins (riboflavin 2 μM, pantothenic acid 2 μM, pyridoxine 0.1 μM and thiamine 0.1 μM) for 12 h (supplementation phase). Germlings were extensively washed with water before shifting to vitamin-starved minimal medium for 36 h (starvation phase). Finally, the cultures were either vitamin supplemented (resupplementation phase) or not supplemented (continued starvation phase) for another 12 h. Dry weight was determined from freeze-dried mycelia.

### *Identification of* A. fumigatus *flavoproteins from the proteome*

For screening flavin-dependent *A. fumigatus* proteins, the *A. fumigatus* Af293 proteome data set (AspGD; http://www.aspergillusgenome.org/download/domains/A_fumigatus_Af293/) from the *Aspergillus* database [19] was searched with the terms ”flavo”, ”flavin”, ”FMN” and ”FAD”. The resulting list was compared with *A. fumigatus* proteins identified in AspGD (http://www.aspgd.org) with terms ”flavo”, ”flavin”, ”FMN” and ”FAD” [20].

### Pulmonary and systemic mouse infection

Three immunocompromised murine models for invasive aspergillosis were used: (i) non-neutropenic model; six-week-old female ICR mice were immunocompromised by subcutaneous injection with cortisone acetate (300 mg/kg) 3 days prior to infection, on the day of infection, and 3, 7, and 11 days postinfection. Fungal strains were grown for 3 days at 37°C on MM agar with appropriate vitamin supplementation. Spores were collected in PBS with 0.2% Tween 20 and counted by a hemocytometer. For the Δ*riboB* strain, conidial viability and germination were not affected on MM supplemented with 2.5 µM riboflavin (Supplementary Figure 3). The mice were infected intranasally with 5 × 10^5^ dormant spores, suspended in 20 μl of PBS plus 0.2% Tween 20 (10 μl in each nostril). Neutropenic (ii) pulmonary and (iii) disseminated models; six-week-old female ICR mice were immunocompromised with cyclophosphamide (150 mg/kg in PBS) injected intraperitoneally at 3 days prior to and at 2 days post-conidial infection. Cortisone acetate (150 mg/kg PBS with 0.1% Tween 80) was injected subcutaneously at 3 days prior to conidial infection. For pulmonary infection, mice were infected intranasally as described above. For disseminated infection, 5 × 10^5^ dormant spores were injected through the lateral tail vein. Survival was monitored for up to 21 days. For histopathology, mice were sacrificed two days after infection, their lungs were removed and stained with Grocott’s methenamine silver stain (GMS; fungal staining) and hematoxylin and eosin (H&E; tissue and nuclear staining). For fungal burden, infected mice were sacrificed on the second day post- infection, their lungs were removed and homogenized, and the homogenates were plated on YAG medium supplemented with the appropriate vitamin for colony forming unit (CFU) enumeration. Experiments were ethically approved by the Ministry of Health (MOH) Animal Welfare Committee, Israel.

## Results

### *Generation of vitamin B auxotrophic mutant strains in* A. fumigatus

To analyse the role of defined vitamin B biosynthetic pathways of *A. fumigatus*, we first identified by bioinformatics analysis, genes encoding key enzymes of the respective pathways for the synthesis of riboflavin (*riboB/Afu1g13300*, encoding GTP cyclohydrolase II), pantothenic acid (*panA/Afu5g11040*, encoding pantoate-beta-alanine ligase), pyridoxine (*pyroA/Afu5g08090*, encoding pyridoxal 5-phosphate synthase) and thiamine (*thiB/Afu2g08970*, encoding thiamine-phosphate diphosphorylase and hydroxyethylthiazole kinase) (Supplementary Figure 1 and Supplementary Tables 3–6). These genes were deleted in the *A. fumigatus akuA::loxP* recipient strain derived from ATCC46645 (AfS77, termed wt here), largely lacking non-homologous recombination [21,22], by homologous recombination and hygromycin selection as described in the Materials and Methods (Supplementary Figure 2). To confirm gene deletion-specific phenotypes, mutant strains were complemented (^C^ strains) by re-integration of functional gene copies. Notably, we also attempted to generate a riboflavin auxotroph by deletion of *Afu2g16360*, the ortholog of *Histoplasma capsulatum rib2*, which is essential for riboflavin biosynthesis in this dimorphic fungal species [23]. Surprisingly, deletion of *Afu2g16360/rib2* did not result in riboflavin auxotrophy in *A. fumigatus* (data not shown), most likely because of the presence of paralogous genes (Supplementary Figure 1A).

### *Deletion of* riboB, panA, pyroA *and* thiB *leads to auxotrophy*

**Figure 1.**
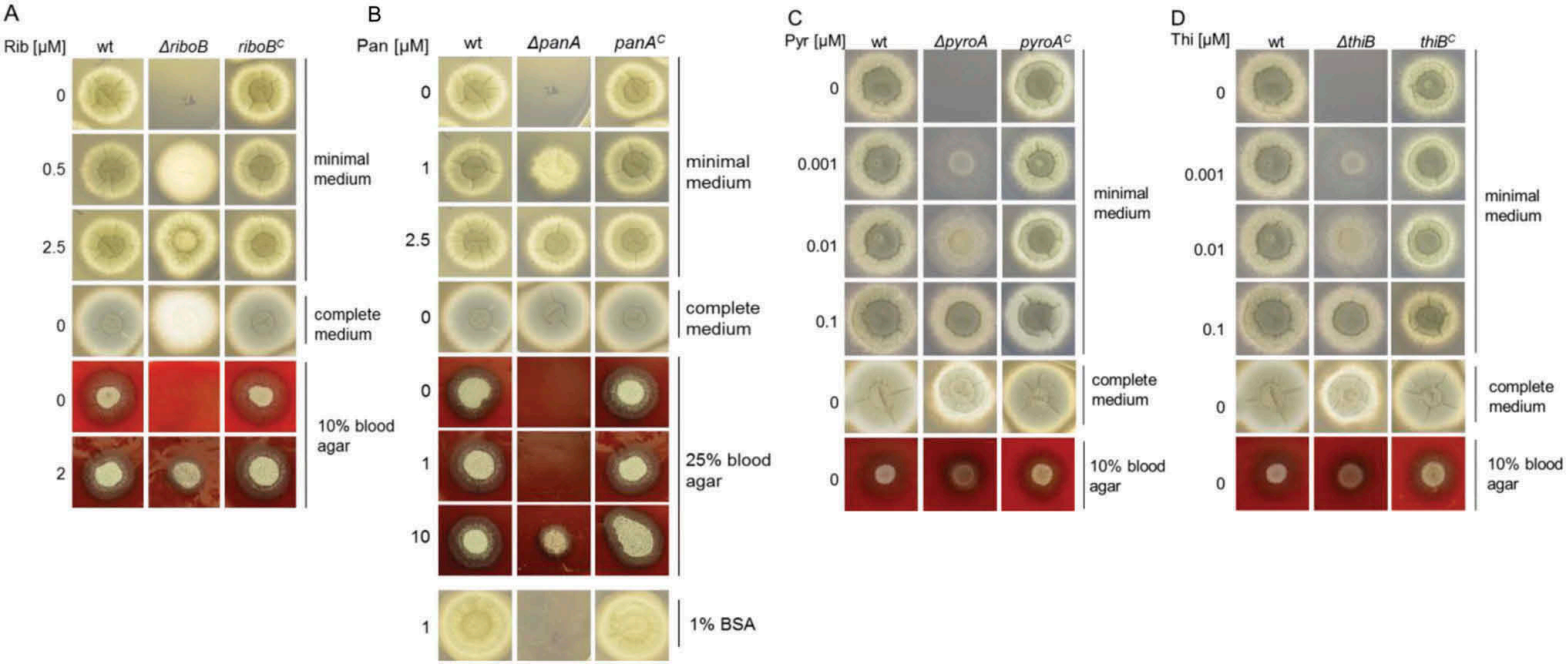
Deletion of *riboB, panA, pyroA* and *thiB* leads to respective auxotrophy. Wild-type AfS77 (wt) and deletion strains (a) *ΔriboB*, (b) *ΔpanA*, (c) *ΔpyroA* and (d) *ΔthiB* and complemented strains *riboB^C^, panA^C^, pyroA^C^* and *thiB^C^* were point-inoculated on agar containing either minimal medium, complete medium or blood. The strains were grown at 37°C for 48 h in the presence of increasing vitamin concentrations. Deletion strains grew only when supplemented with their respective vitamin, whereas wt and complemented strains did not require supplementation.

The growth of the mutant strains was compared to the wt and complemented strains by spotting 10^4^ conidia on solid minimal medium, complete medium or blood agar, containing different concentrations of vitamin as shown in Figure 1. No visible growth was seen in the mutant strains in the absence of their respective vitamin supplementation, confirming that the gene deletions resulted in auxotrophy. Radial growth of the Δ*riboB* and Δ*panA* mutants was fully restored by addition of 2.5 µM riboflavin or pantothenic acid (Figure 1(a,b)), respectively, whereas growth of the *ΔpyroA* and *ΔthiB* strains necessitated addition of only 0.1 µM of their respective vitamin (Figure 1(c,d)). Complemented strains showed wild-type phenotypes in all assays performed, confirming the specificity of gene deletion phenotypes (Figure 1). Complete medium allowed full growth and sporulation of the Δ*panA, ΔpyroA* and *ΔthiB* strains, whereas in Δ*riboB* conidiation was blocked (reflected by the white instead of greenish colonies), most likely because the amount of riboflavin in the medium, which derives from the yeast extract component in this medium, is not sufficient to support wt-like growth for ∆*riboB*.

In contrast to the *ΔpyroA* and *ΔthiB* strains, both Δ*riboB* and Δ*panA* did not grow on blood agar without vitamin supplementation, indicating that blood riboflavin and pantothenic acid levels are too low to support their growth (Figure 1(a–d)). Interestingly, for growth of Δ*panA* on blood agar, increased pantothenic acid supplementation (10 µM) was required. This effect is most likely because pantothenic acid is adsorbed by serum albumin in the blood. In agreement, 1% bovine serum albumin (BSA) blocked growth of Δ*panA* in the presence of 1 µM pantothenic acid in minimal medium, which supports growth without BSA (Figure 1(b)).

**Figure 2.**
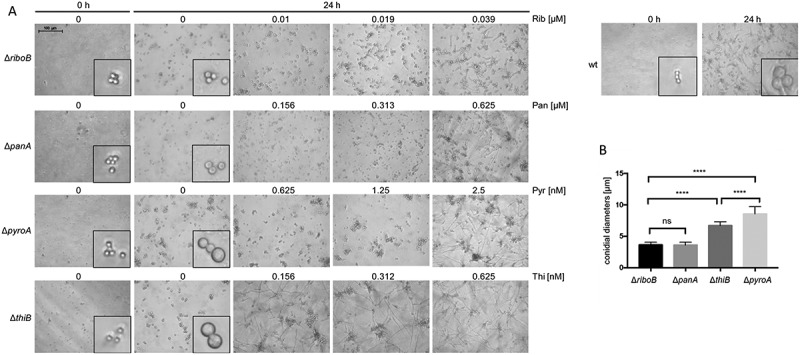
Microscopic analysis of germination and growth of the deletion strains. (a) Wild- type AfS77 (wt) and deletion strains *ΔriboB, ΔpanA, ΔpyroA* and *ΔthiB* were grown in minimal liquid medium at 37°C for 24 h in the presence of increasing vitamin concentrations and analyzed by light microscopy. Higher magnification (insets, left panel) demonstrated significant swelling of *ΔpyroA* (P < 0.0001 *vs. ΔriboB* and *ΔpanA*) and especially *ΔthiB* conidia (P < 0.0001 vs. *ΔriboB* and *ΔpanA*) in the absence of vitamin supplementation compared to freshly harvested ungerminated conidia (0 h). (b) Quantification of conidial diameter of the deletion strains grown in liquid minimal medium at 37°C for 24 h in the absence of the respective vitamin.

In liquid minimal medium without vitamin, after 24 h at 37°C, Δ*riboB* and Δ*panA* conidia remained compact, whereas *ΔpyroA* and especially *ΔthiB* conidia underwent significant swelling, indicating partial early germination (Figure 2(a), insets, Figure 2(b)). Interestingly, in liquid minimal medium, vitamin supplementation restored mutant growth at concentrations far lower than those needed on minimal medium agar plates (Figure 2).

### Riboflavin and its derivatives FAD and FMN play a crucial role in NO detoxification and sporulation

**Figure 3.**
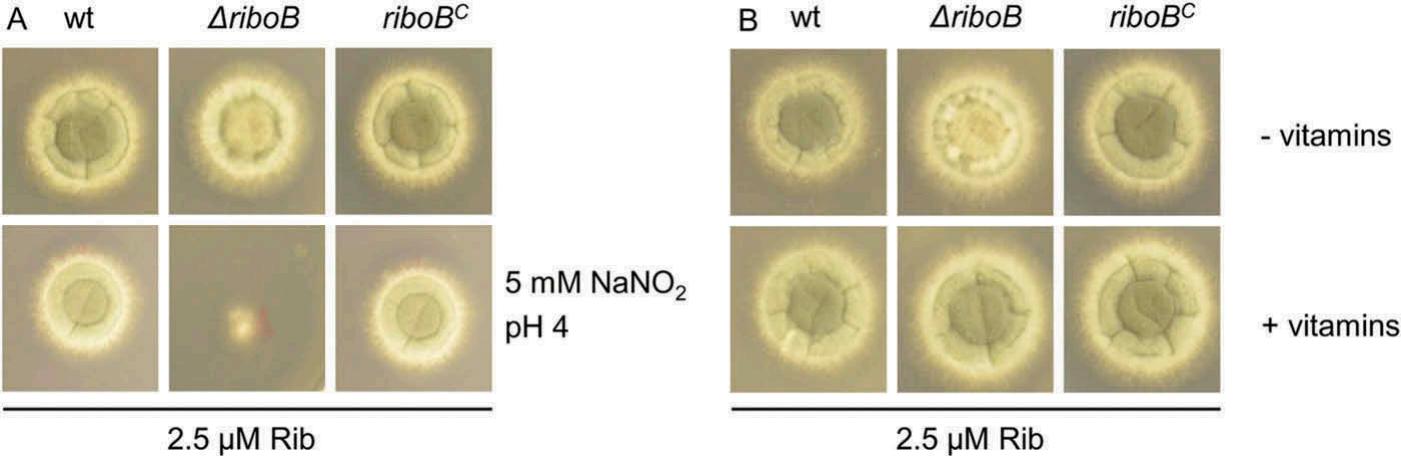
Riboflavin and its derivatives FAD and FMN play a crucial role in NO detoxification and sporulation. Wild-type AfS77 (wt), *ΔriboB* deletion and complemented *riboB^C^* strains were point-inoculated on minimal medium agar and grown at 37°C for 48 h in the presence of 2.5 µM riboflavin. Addition of (a) 5 mM nitrite (NaNO_2_) selectively inhibited the growth of *ΔriboB*. (b) Supplementation with pantothenic acid, pyridoxine and niacin (+ vitamins) restored conidiation in *ΔriboB* reflected by the restoration of the greenish colony pigmentation.

Riboflavin is the precursor of flavin adenine dinucleotide (FAD) and flavin mononucleotide (FMN) and as such used by approximately 238 flavoproteins in *A. fumigatus* (Supplementary Table 7). Flavoproteins have central metabolic and regulatory roles; Table 1 displays 22 pathways comprising 91 of the 238 *A. fumigatus* flavoproteins. FAD is a crucial coenzyme of flavohemoglobins, which provide protection against NO and related reactive nitrogen species [24]. We therefore tested the growth of Δ*riboB* during NO stress, induced by addition of 5 mM NaNO_2_ at a low pH of 4.0 (acidic conditions stimulate generation of nitric oxide from nitrate [25]), and limiting 2.5 µM riboflavin for growth. Growth of the mutant was inhibited compared to the wt and complemented strains, indicating an important role for riboflavin in nitric oxide (NO) detoxification (Figure 3(a)). FAD is also a crucial coenzyme for the biosynthesis of the B vitamins niacin, pantothenic acid and pyridoxine. We therefore tested whether we can correct the impaired sporulation of Δ*riboB* on minimal medium (reflected by a decrease in greenish pigmentation of the colony) in the presence of 2.5 µM riboflavin, by adding these vitamins on minimal medium. We found that supplementation of Δ*riboB* with 2.5 µM pantothenic acid, 10 µM pyridoxine and 10 µM niacin restored conidiation, suggesting a role of riboflavin in biosynthesis of these vitamins (Figure 3(b), Table 1).

The vitamins riboflavin, pantothenic acid, pyridoxine and thiamine have potent anti- oxidative activity. We therefore tested the sensitivity of the Δ*riboB*, Δ*panA, ΔpyroA* and *ΔthiB* strains to survive oxidative stress induced by H_2_O_2_ or menadione under partial supplementation with their respective vitamin. However, we found no differences in the sensitivity of the mutants compared to the wt (data not shown).

### *Decreased riboflavin availability reduces production of siderophores in* A. fumigatus

**Figure 4.**
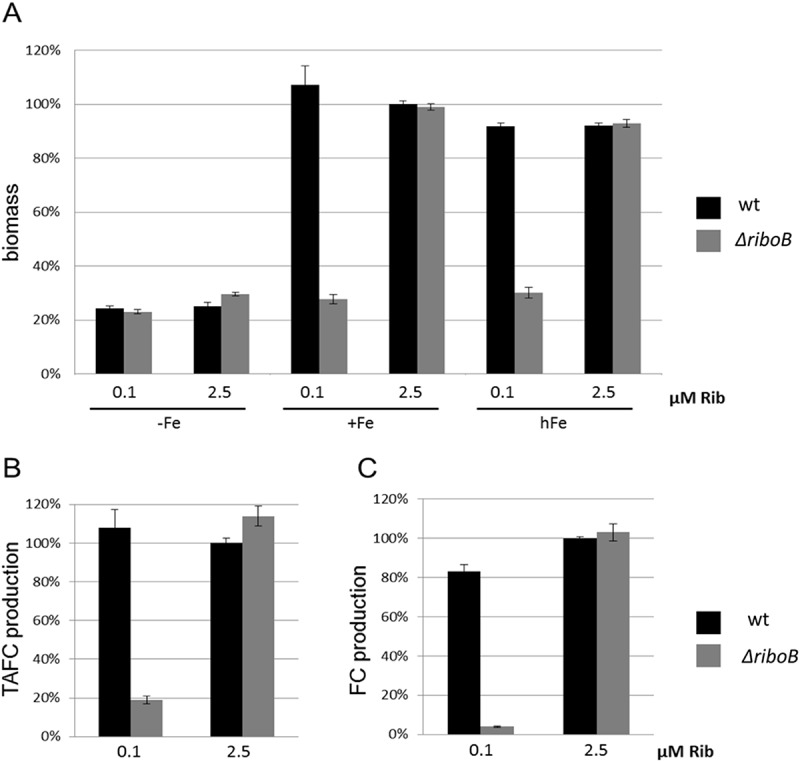
Decreased riboflavin availability reduces production of siderophores in *A. fumigatus.* (a) Wt and Δ*riboB* strains were grown for 24 h at 37°C in liquid minimal medium under iron starvation (-Fe), iron sufficiency (+Fe, 30 µM FeSO_4_) and iron excess (hFe, 5 mM FeSO_4_) supplemented with either a low (0.1 µM) or a high (2.5 µM) riboflavin (Rib) concentration and their dry weight assessed. Under iron sufficiency and iron excess and low riboflavin supplementation of 0.1 µM, Δ*riboB* displayed a significantly decreased biomass production (+Fe: P < 0.0001 vs. wt; hFe: P < 0.0001 vs. wt) compared to the wt and to the mutant´s biomass production with high riboflavin supplementation. During iron starvation and low riboflavin supplementation, Δ*riboB* displayed markedly reduced (b) TAFC extracellular siderophore (P < 0.001 vs. wt) and (c) FC intracellular siderophore production (P < 0.001 vs. wt).

To survive in the human host under conditions of iron starvation, *A. fumigatus* employs two high-affinity iron-uptake systems, reductive iron assimilation and the extracellular siderophores fusarinine C (FsC) and triacetylfusarinine C (TAFC), as well as the intracellular siderophore ferricrocin (FC) for iron storage and distribution [26,27]. To further characterize the role of riboflavin biosynthesis in iron utilization and siderophore production, wt and Δ*riboB* were analysed after growth in liquid minimal medium during iron starvation (-Fe), iron sufficiency (+Fe, 30 µM FeSO_4_) and iron excess (hFe, 5 mM FeSO_4_) supplemented with either a low (0.1 µM) or a high (2.5 µM) riboflavin concentration (Figure 4). Biomass production was significantly decreased in Δ*riboB* compared to wt under low riboflavin and iron sufficiency (74% less, P < 0.0001 vs. wt) or iron excess (67% less, P < 0.0001 vs. wt) (Figure 4(a)). Siderophore biosynthesis was not observed in the wt or Δ*riboB* under +Fe or hFe (data not shown). During iron starvation, biomass production of the mutant displayed wt-like levels even under the low riboflavin concentration (Figure 4(a)), most likely due to the approximately fourfold decrease in biomass compared to iron sufficiency or excess that results in decreased vitamin requirement for growth. Interestingly, at low (0.1 µM) riboflavin concentration under iron starvation, siderophore production in Δ*riboB* decreased significantly: 82% less TAFC (P < 0.0001 vs. wt) and 96% less FC (P < 0.0001 vs. wt) compared to the wt, despite the fact that under these conditions both strains achieve the same biomass (Figure 4(b,c). These findings indicate a crucial role for riboflavin in siderophore biosynthesis, most likely as a cofactor for ornithine-N^5^-monooxygenase SidA (Table 1), which catalyzes the initial step in siderophore biosynthesis [28].

### *Iron supplementation reduces pantothenic acid requirement of*ΔpanA

**Figure 5.**
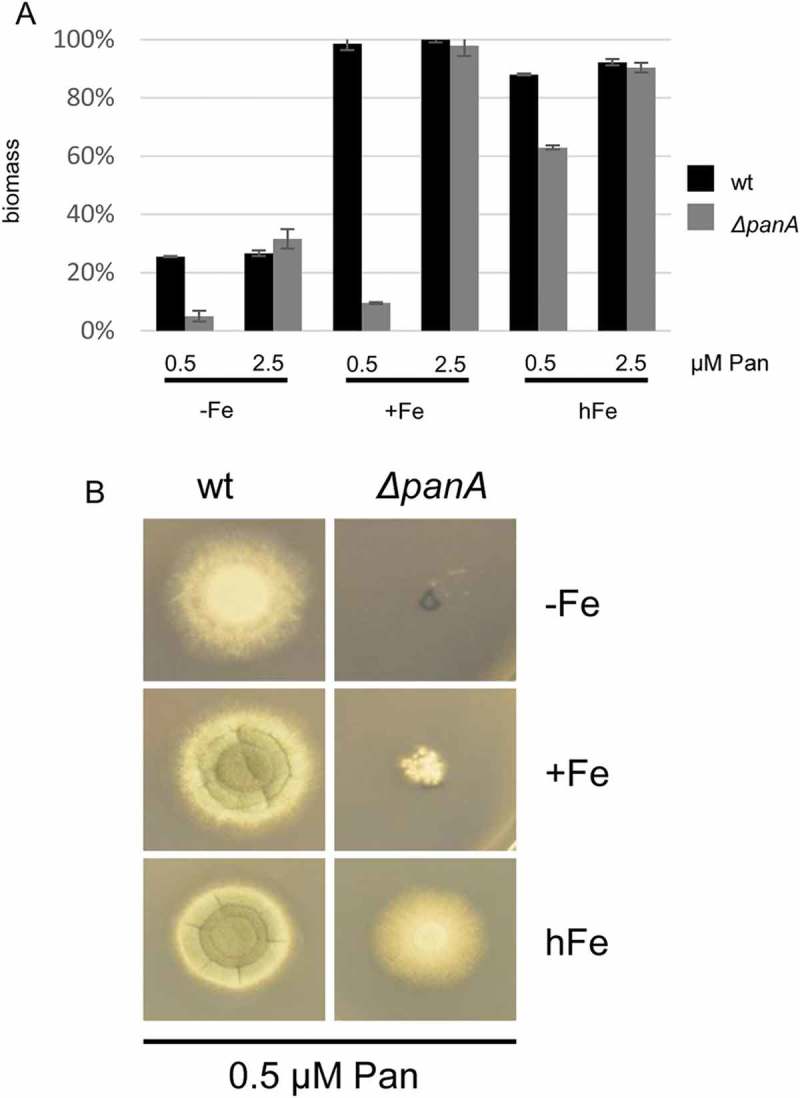
Iron supplementation reduces pantothenic acid requirement of Δ*panA.* (a) Wt and Δ*panA* strains were grown for 24 h at 37°C in liquid minimal medium under either a low (0.5 µM) or a high (2.5 µM) pantothenic acid (Pan) concentration. Iron excess (hFe, 5 mM FeSO_4_) significantly increased Δ*panA* biomass compared to growth under iron sufficiency (+Fe, 30 µM FeSO_4_) during low riboflavin supplementation (P < 0.0001 vs. +Fe). (b) Increased iron concentrations partially corrected Δ*panA* radial growth under low (0.5 µM) pantothenic acid supplementation on minimal medium agar.

Siderophore biosynthesis involves the enzymatic activity of nonribosomal peptide synthetases (NRPS), which contain 4´-phosphopantetheine, derived from pantothenic acid, as an essential prosthetic group. In *A. fumigatus* one such enzyme is encoded by *npgA/pptA*. Previous studies confirmed that NpgA function is essential for biosynthesis of TAFC and FC [29] and consequently for iron homeostasis. To characterize the effects of PanA deficiency on iron homeostasis, we analyzed the biomass production of the pantothenic acid auxotrophic mutant strain Δ*panA* relative to the wt strain under iron depleted (-Fe), iron replete (+Fe, 30 µM FeSO_4_) and iron excess (hFe, 5 mM FeSO_4_) conditions, with either low (0.5 µM) or high (2.5 µM) concentrations of pantothenic acid. Under -Fe and +Fe conditions, Δ*panA* exhibited significantly decreased biomass production of 80% during -Fe (P < 0.001 vs. wt) and 90% during +Fe (P < 0.0001 vs. wt) when supplemented with the low pantothenic acid concentration (while biomass production under high iron conditions was only mildly reduced by 28% compared to the wt). However, wt-like growth of the mutant strain was achieved by the addition of 2.5 µM pantothenic acid into the medium (Figure 5(a)). The beneficial effect of iron excess on biomass production of the pantothenic acid auxotroph was also demonstrated on solid medium (Figure 5(b)). In contrast, iron excess did not enhance growth of the *riboB, pyroA* and *thiB* null strains under limiting vitamin supplementation, further indicating the specificity of this effect in the *ΔpanA* mutant (data not shown). These results suggest a role for pantothenic acid in iron metabolism. In agreement, pantothenic acid is essential for activation of nonribosomal peptide synthetases, including siderophore biosynthetic SidC and SidD [30,31].

### *Hypoxia decreases pantothenic acid requirement of*ΔpanA

**Figure 6.**
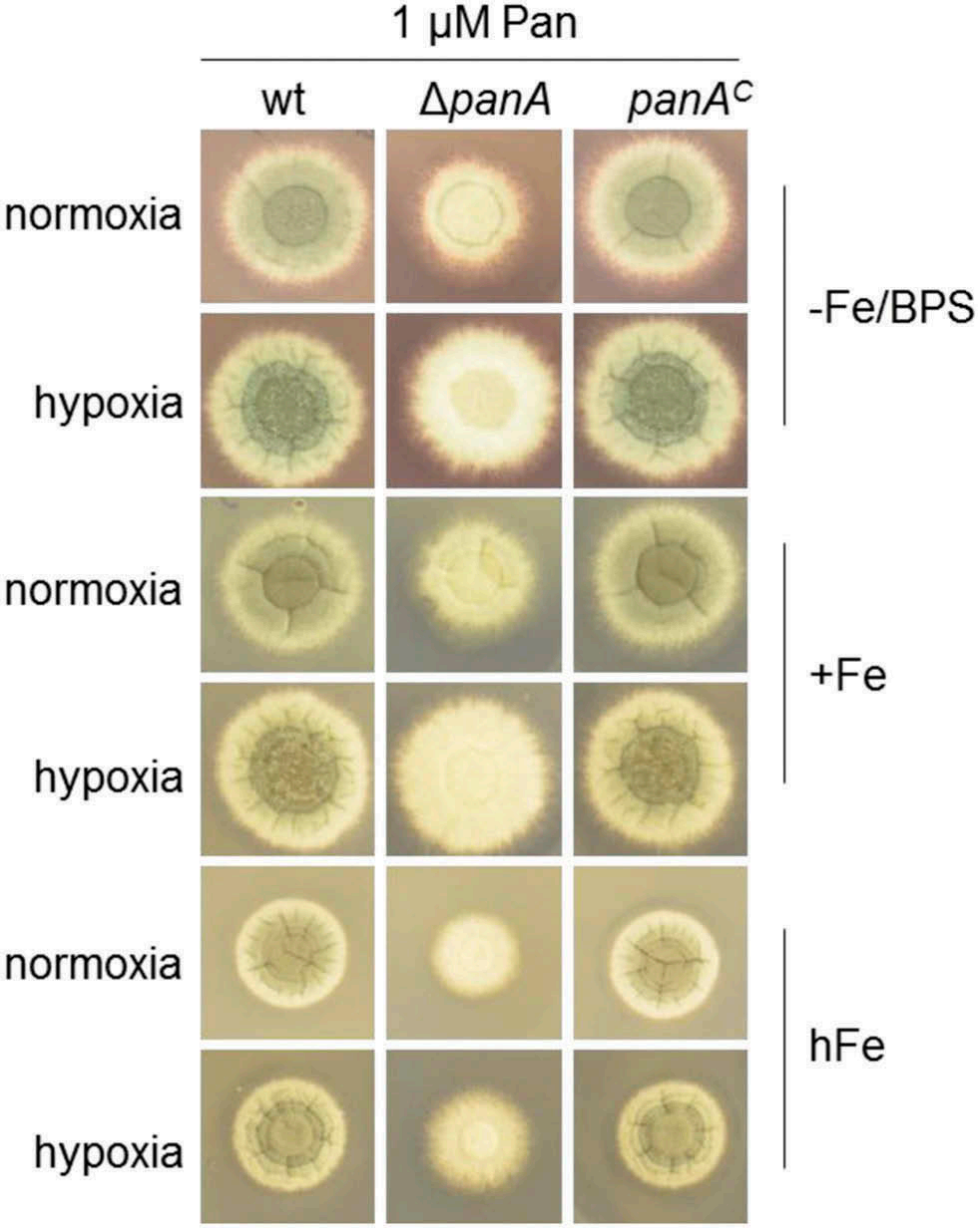
Hypoxia decreases pantothenic acid requirement of Δ*panA.* Wt, Δ*panA* and complemented *panA^C^* strains were point inoculated on minimal medium agar containing low (1 µM) pantothenic acid (Pan). Strains were grown for 48 h at 37°C under iron starvation (-Fe/BPS iron chelator), iron sufficiency (+Fe, 30 µM FeSO_4_) or iron excess (hFe, 5 mM FeSO_4_). Hypoxia markedly increases Δ*panA* radial growth under iron starvation (-Fe/BPS) and iron sufficiency (+Fe) compared to normoxia. Δ*panA* conidiation was strongly inhibited during hypoxia at all iron concentrations.

To survive in the hypoxic necrotic tissue during infection, *A. fumigatus* is able to adapt to extremely low oxygen and low iron surroundings. On minimal medium agar plates, the growth defect of Δ*panA* supplemented with limiting 1 µM pantothenic acid was less pronounced under hypoxic (1% oxygen) conditions compared to normoxic (21% oxygen) conditions during iron starvation (-Fe), iron sufficiency (+Fe) and iron excess (hFe, 5 mM FeSO_4_) (Figure 6). The most remarkable phenotype was observed during iron starvation and iron sufficiency, where the Δ*panA* strain under hypoxic conditions reached radial wt-like growth but completely lacked sporulation. The role of pantothenic acid during hypoxic conditions could be explained by reduced pantothenic acid requirement or by increased pantothenic uptake under these conditions [32]. In contrast, the *riboB, pyroA and thiB* null strains under limiting vitamin supplementation and normoxic or hypoxic iron-sufficient conditions grew like the wt (data not shown).

### RiboB, panA, pyroA *and* thiB *deletion mutants are not killed by vitamin starvation*

**Figure 7.**
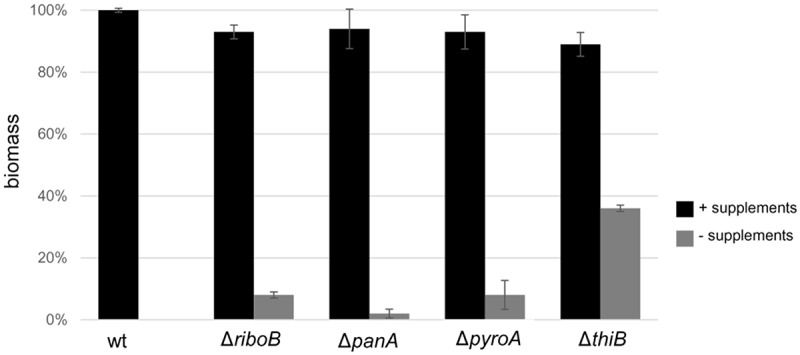
*riboB, panA, pyroA* and *thiB* deletion mutants are not killed by starvation of the lacking vitamin. Deletion strains *ΔriboB, ΔpanA, ΔpyroA* and *ΔthiB* were grown in vitamin-supplemented liquid minimal medium for 12 h, washed and vitamin-starved for 36 h and finally either vitamin-re-supplemented (black bars) or not supplemented (grey bars) for another 12 h. Wild-type AfS77 (wt) received the same treatment but was nitrogen-starved (instead of vitamin-starved as in the case of auxotrophic mutants) by growing without the sole nitrogen source glutamine in the 36 h starvation phase and either glutamine re-supplemented (black bars) or not supplemented for another 12 h. The biomass of the mutants was normalized to that of the wt. Biomass measurements at the end of the experiment indicate that following vitamin supplementation, the four auxotrophs remained viable and recovered their growth (black bars).

To evaluate the suitability of RiboB, PanA, PyroA and ThiB as possible drug targets, we carried out vitamin-shift experiments to measure whether growth in the absence of vitamin causes growth arrest (*i.e*. inhibiting the pathway is fungistatic) or cell death (*i.e*. inhibiting the pathway is fungicidal). Auxotrophic strains were germinated in liquid minimal medium for 12 h in the presence of the respective vitamin (corresponding to early wt growth in the lungs during infection), and then washed repeatedly and grown for 36 h in the absence of the vitamin (corresponding to inhibition of the pathway). Finally, to determine viability, the vitamin was added to the medium for 12 h and the dry weight of the lyophilized mycelium was measured relative to non-supplemented strains. For comparison, the wt strain was starved for nitrogen (instead of vitamins as in the case of the auxotrophic mutants) and re-supplemented with nitrogen for 12h, assuming that wt strains are capable of coping with nitrogen starvation conditions. We found that the vitamin–starved mycelia of the Δ*riboB*, Δ*panA*, Δ*pyroA* and Δ*thiB* strains grew rapidly when re-supplemented with their respective vitamin, comparable to the nitrogen-starved wt (Figure 7, black bars), while without supplementation, mycelial growth remained minimal (Figure 7, grey bars). This indicates that the mycelium remained alive during the 36 h of vitamin-starvation and suggests that inhibition of these pathways will have a fungistatic (and not a fungicidal) effect.

### *Riboflavin and pantothenic acid biosynthesis are required for* A. fumigatus *virulence in murine infection models*

**Figure 8.**
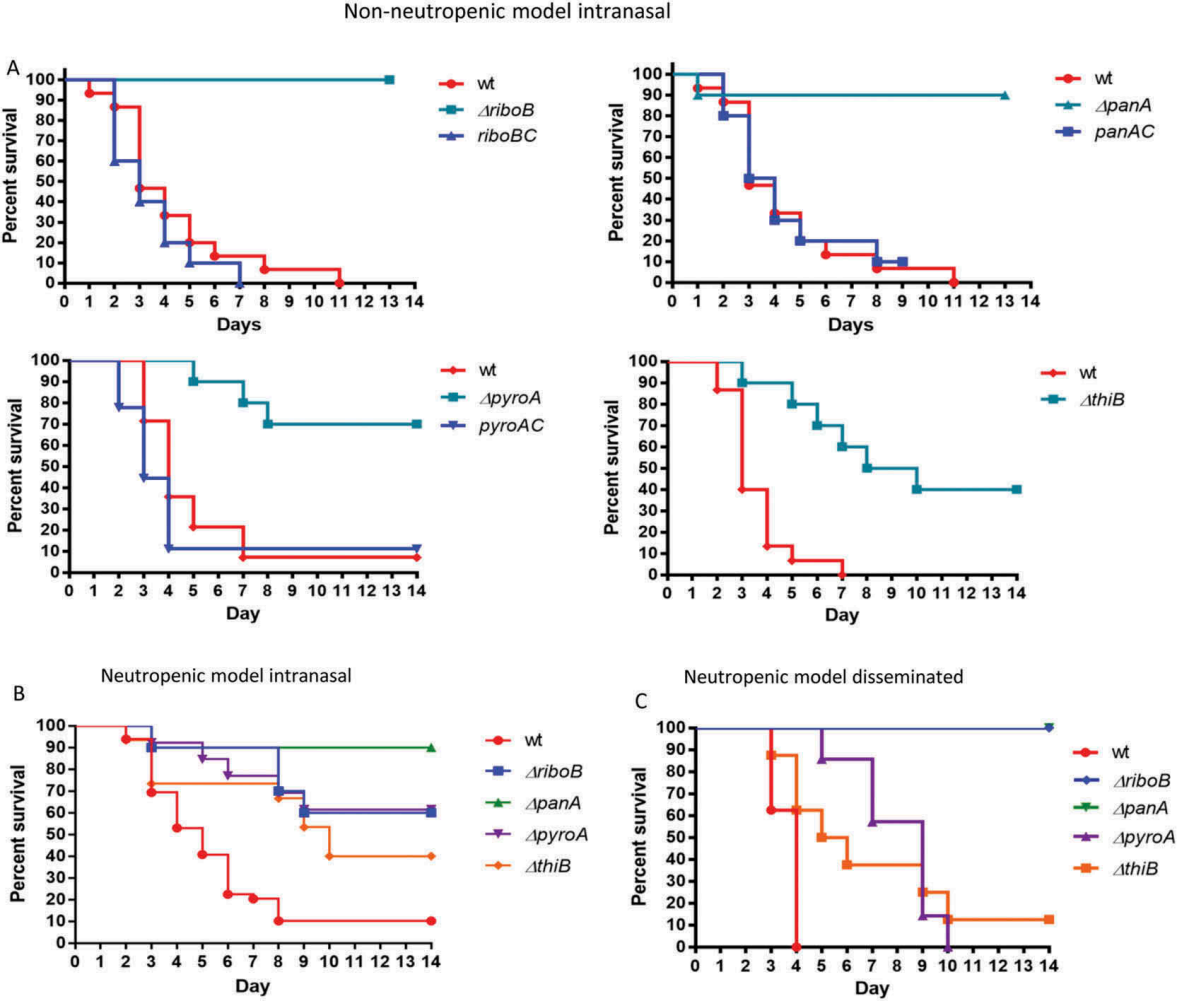
Riboflavin and pantothenic acid biosynthesis are essential for virulence of *A. fumigatus* in murine infection models. Mouse survival curves following intranasal infection of (a) cortisone-acetate immunocompromised mice (n = 10 animals/group) and (b) cyclophosphamide-immunocompromised neutropenic mice (wt, n = 49; *ΔriboB*, n = 10; *ΔpanA*, n = 10; *ΔpyroA*, n = 13; *ΔthiB*, n = 15 animals/group). (c) Intravenous disseminated infection of cyclophosphamide-immunocompromised neutropenic mice, wt, n = 8; *ΔriboB*, n = 5; *ΔpanA*, n = 5; *ΔpyroA*, n = 8; *ΔthiB*, n = 8 animals/group).

**Figure 9.**
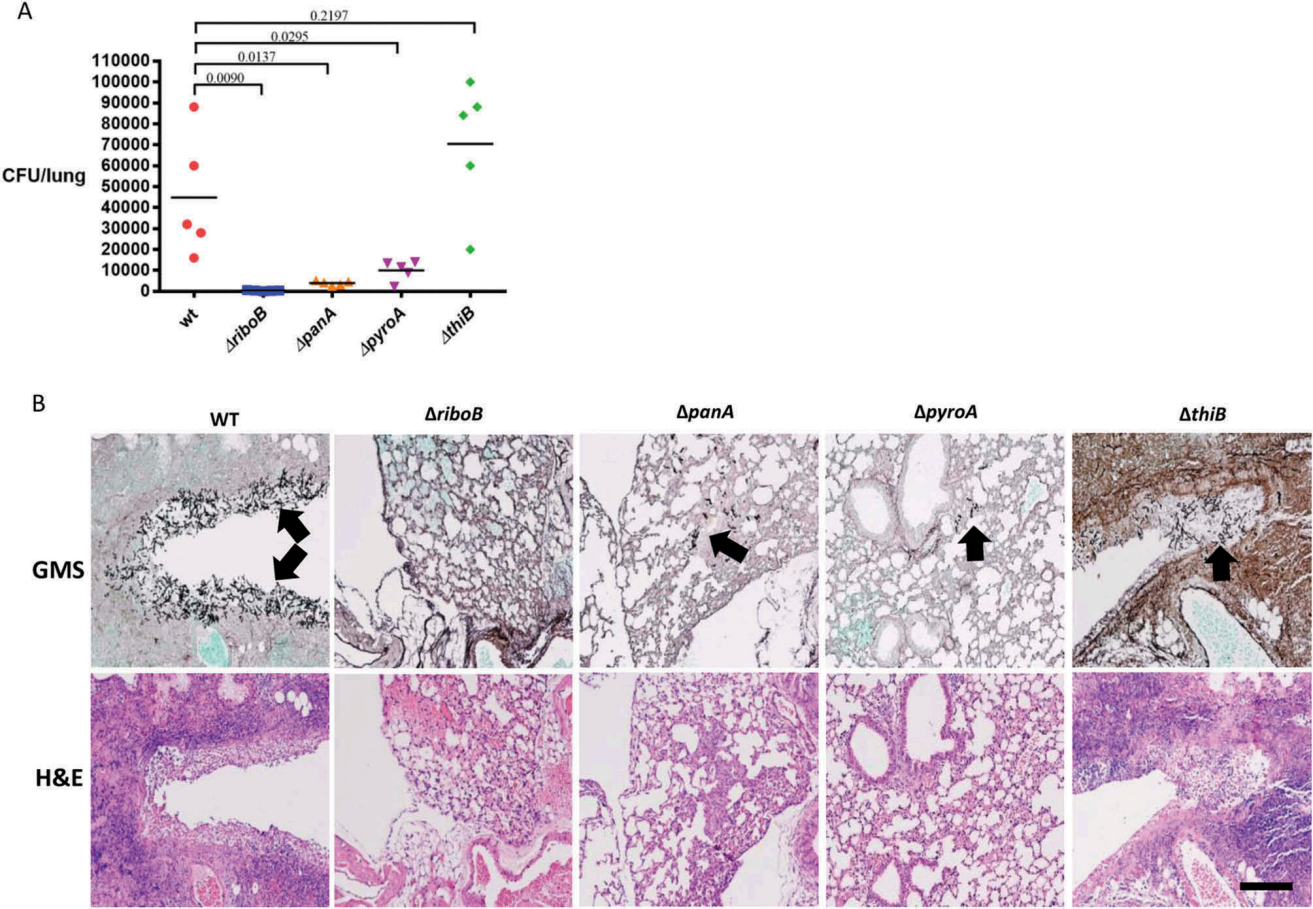
Deficiency in riboflavin, pantothenic acid and pyridoxine biosynthesis significantly reduce *A. fumigatus* lung burden in cortisone-acetate immunocompromised mice. (a) Lung fungal load and (b) lung histology of intranasally-infected cortisone-acetate immunocompromised mice (n = 5 animals/group), after 48 h of infection. Staining was done with GMS (stains fungus black, see black arrows) or H&E lung stain. Bar = 200 µm.

The Δ*riboB*, Δ*panA*, Δ*pyroA*, Δ*thiB* strains were assessed for virulence in three immunocompromised murine models of infection: (i) a non-neutropenic pulmonary infection model in which mice were immunosuppressed with cortisone acetate (CA), (ii) a neutropenic pulmonary infection model in which mice were immunosuppressed with cyclophosphamide (CY), (iii) a neutropenic disseminated infection model in which mice were immunosuppressed with cyclophosphamide (CY) and infected via the lateral tail vein. The Δ*riboB* mutant was avirulent (100% survival) and Δ*panA* mutant was strongly attenuated (90% survival) in the non-neutropenic pulmonary model of infection, both strains were avirulent (100% survival) in the disseminated neutropenic model of infection (P < 0.0001 vs. wt), and strongly attenuated in virulence (90% survival Δ*panA*, 60% survival Δ*riboB*) in the neutropenic pulmonary model of infection (P < 0.0005 vs. wt) (Figure 8(a–c)). The Δ*pyroA* and Δ*thiB* mutants showed attenuated virulence in both pulmonary models of infection (60 to 70% survival Δ*pyroA*, 40% survival Δ*thiB*, P < 0.005 vs. wt) and in disseminated infection (P < 0.05 vs. wt) although in this last model 100% mortality of Δ*pyroA* and 90% mortality of Δ*thiB* was observed, possibly suggesting that there are lower levels of available pyridoxine and thiamine in the lungs compared to blood (Figure 8(a–c)). It should be noted that in the neutropenic models, profound neutropenia resolves after ~ 7 days and that, had an additional round of immunosuppression been administered at day + 6, the virulence data may have been slightly less compelling for some of the strains. Fungal load and histopathology of infected lungs (CA, non-neutropenic host model) further reflected these results. No CFUs or visible fungal growth were found in the lungs of mice infected for 48 h with the Δ*riboB* strain. Low lung CFU counts were found in Δ*panA* (~ 2% of wt), increasing in Δ*pyroA* (~ 20% of wt), and reaching wt levels in Δ*thiB* (Figure 9(a)). Histopathology followed by GMS (stains fungal elements black) and H&E (stains lung tissue) further corroborated these findings, showing extensive hyphal growth (arrows) and granulocyte infiltration (purple granulation-lower panel) in the airways of mice infected with the wt strain, which was slightly attenuated following Δ*thiB* infection, greatly reduced following Δ*pyroA* and Δ*panA* infection and totally absent in the Δ*riboB*–infected mice (Figure 9(b)).

## Discussion

A central problem in the development of antifungals is the lack of suitable drug targets not found in humans. In this report, using the pathogenic mold *A. fumigatus* as our model, we analysed four fungal-specific pathways involved in the biosynthesis of riboflavin (vitamin B2), pantothenic acid (vitamin B5), pyridoxine (vitamin B6) and thiamine (vitamin B1) as potential drug targets.

We deleted genes encoding key enzymes in each of these pathways (*riboB, panA, pyroA* and *thiB*), which resulted in auxotrophy. Our underlying hypothesis was that the vitamins produced by these pathways might not be available for fungal growth in the host, resulting in dependence on endogenous biosynthesis. Past work in *A. fumigatus* has shown this to be the case for folate (Vitamin B9) biosynthesis. Deletion of *pabA*, encoding PABA synthase, catalysing an early step in folate biosynthesis, abrogated virulence in infected mice, designating this pathway as an excellent candidate for the development of novel antifungals [33]. Antibacterial sulfa drugs that inhibit dihydropteroate synthase in the folic acid biosynthetic pathway already exist and show weak antifungal activity [34]. Recent efforts have focused on developing antifungals to target other enzymes in this pathway [35].

Our main finding was that deletion of *riboB* and *panA* abolished or strongly attenuated virulence (depending on the infection model), *pyroA* deletion attenuated virulence and loss of *thiB* weakly reduced virulence. Virulence was tested in three models of murine infection that recapitulate the most common types of invasive aspergillosis infections [36]. We modelled invasive pulmonary aspergillosis in the neutropenic host using cyclophosphamide and in the non-neutropenic compromised host using cortisone acetate. Disseminated aspergillosis, which frequently occurs in the neutropenic host, was performed by intravenous infection. Our results suggest that there are insufficient levels of riboflavin and pantothenic acid in the infected host to support growth of the Δ*riboB* and Δ*panA* mutants. In contrast, the Δ*pyroA* and Δ*thiB* mutants need only very low levels (0.1–2.5 nM) of external pyridoxine and thiamine for growth, which are available in the host at far higher concentrations [37,38], most likely explaining their milder influence on virulence. Notably, the virulence behaviour of the four mutants matched their growth pattern on blood agar plates: in contrast to the Δ*pyroA* and Δ*thiB* strains, Δ*riboB* and Δ*panA* mutant strains were unable to grow on blood agar without vitamin supplementation. Virulence studies with auxotrophic mutants also serve to describe nutrient availability in different host niches. The strongly attenuated virulence of Δ*riboB* and Δ*panA* mutants in all tested virulence models demonstrates that both riboflavin and pantothenic acid biosynthesis are not only essential for invasion (intranasal infection model) but also during dissemination (intravenous infection model). In contrast, lysine biosynthesis was previously found to be essential exclusively for invasion but not during dissemination [10,11]. Virulence analysis of auxotrophs was previously tested in the closely related *Aspergillus nidulans* during the 1970s. Interestingly, they gave slightly different results. As found here for *A. fumigatus*, thiamine auxotrophy did not reduce virulence. However, in *A. nidulans*, riboflavin auxotrophy attenuated but did not abolish virulence whereas pyridoxine auxotrophy abolished virulence [39]. However, these studies were performed with mutagenized strains in which the target genes were not deleted and they lacked the complemented strains as controls.

Riboflavin is the precursor of flavin mononucleotide (FMN) and flavin adenine nucleotide (FAD) and as such is an essential cofactor for many flavoprotein-catalyzed physiological processes. Remarkably, *A. fumigatus* encodes approximately 238 flavoproteins (Supplementary Table 7) involved in central metabolic pathways and regulatory circuits (Table 1) including for example NO-detoxification (flavohemoglobins FhpA and FhpB), siderophore biosynthesis (SidA), reductive iron assimilation (metalloreductase FreB), amino acid biosynthesis, ergosterol biosynthesis, vitamin biosynthesis, and secondary metabolism. In contrast, *S. cerevisiae* possesses only 68 flavoproteins [40]. The greatly increased number of flavoproteins in *A. fumigatus* compared to *S. cerevisiae* might indicate a higher demand for riboflavin. We confirmed here the importance of riboflavin for NO-detoxification and siderophore biosynthesis in *A. fumigatus*. In contrast to NO-detoxification, which appears to be dispensable for virulence of *A. fumigatus* [41], siderophore biosynthesis has been shown to be essential for virulence of several animal and plant pathogenic fungi [42]. Similarly, pantothenic acid is of central importance for primary metabolism as it is essential for coenzyme A biosynthesis and consequently for fatty acid metabolism, carbohydrate metabolism and tricarboxylic acid cycle [43]. Moreover, it is an essential prosthetic group for polyketide synthases (PKSs) and nonribosomal peptide synthetases (NRPSs) including the siderophore biosynthetic NRPSs. The impact of PKSs and NRPSs is reflected by the avirulence of the *A. fumigatus* mutant lacking the pantothenic acid dependent enzyme phosphopantetheinyl transferase PptA/NpgA [30]. The requirement of PptA/NpgA and consequently pantothenic acid for activation of NRPSs including the siderophore biosynthetic NRPSs SidD and SidC is consistent with the beneficial effect of iron supplementation on biomass production of Δ*panA* during limited pantothenic acid supplementation (Figure 4).

How suitable are the riboflavin and pantothenic acid biosynthetic pathways for development as drug targets? The riboflavin biosynthesis pathway contains many genes absent in humans, including *rib3, rib4, rib5, rib7* and *rib1/riboB* described here (see supplementary Table 3). We show that deletion of *riboB* in *A. fumigatus* leads to avirulence in cortisone-compromised mice and in neutropenic mice with disseminated infection. Lung CFU enumeration and histology show complete clearance of the fungus from the lungs 48 h after infection. Riboflavin auxotrophy also leads to avirulence in *Candida albicans* [6], *Histoplasma capsulatum* [23] and in many species of gram-negative bacteria including Mycobacteria that lack a riboflavin uptake system and are therefore totally dependent on endogenous biosynthesis [44]. Therefore, this pathway is particularly suitable for the development of antimicrobials. Based on detailed enzymatic and structural data, substrate inhibitors have been developed for the last two steps of riboflavin biosynthesis, catalysed by lumazine synthase and riboflavin synthase, respectively. To the best of our knowledge, *in vivo* use of these compounds has not been described, suggesting there were pharmacological problems in their development [45,46].

The pantothenic acid biosynthesis pathway is also rich in possible antimicrobial targets including *pan2, pan5*, and pantothenate synthase *pan6/panA* (see supplementary Table 4) [47]. A major effort has been made to develop pantothenate synthase inhibitors active against *Mycobacterium tuberculosis*, as this enzyme is essential for virulence [48]. Although several showed *in vitro* activity, none was described as acting *in vivo* [49]. Subsequent CoA synthesis from pantothenate is controlled by pantothenate kinase, encoded by the essential gene *cab1* (*S. cerevisiae*)/*panK* (*A. nidulans*) [50]. Several pantothenic acid analogues that inhibit this enzyme have shown antibacterial activity *in vitro* and *in vivo* [47] but none were tested for fungal infections.

Two drawbacks should be noted regarding the suitability of the enzymes RiboB and PanA as antifungal targets- first, our *in vitro* vitamin-shift analysis suggests that inhibiting these enzymes will have a fungistatic rather than fungicidal action. Second, inhibition of a single cellular target often results in mutations leading to resistance. Nevertheless, these problems can be circumvented by developing inhibitors that (i) react with the target enzyme to generate toxic products or (ii) block additional essential fungal targets, and (iii) by using drug combinations [3].

Future work needs to test existing inhibitors of riboflavin and pantothenic acid biosynthesis for activity in pathogenic fungi both *in vitro* and *in vivo*. Crystallographic data of key fungal enzymes (RiboB, PanA etc.) needs to be generated and used to plan more specific and potent antifungal inhibitors. Compound library screens can make use of the fact that inhibitors specifically acting on these pathways are neutralized by the addition of exogenous riboflavin and pantothenic acid, respectively [51].

## References

[1] Brown GD, Denning DW, Levitz SM. Tackling human fungal infections. Science. 2012;336(6082):647 PubMed PMID: 22582229. 22582229 10.1126/science.1222236

[2] Bongomin F, Gago S, Oladele RO, et al Global and multi-national prevalence of fungal diseases-estimate precision. J Fungi (Basel). 2017;3(4). PubMed PMID: 29371573; PubMed Central PMCID: PMCPMC5753159 DOI:10.3390/jof3040057 PMC5753159 29371573

[3] Perfect JR The antifungal pipeline: a reality check. Nat Rev Drug Discov. 2017;16(9):603–616. PubMed PMID: 28496146. 28496146 10.1038/nrd.2017.46 PMC5760994

[4] Lu Y, Deng J, Rhodes JC, et al Predicting essential genes for identifying potential drug targets in *Aspergillus fumigatus*. Comput Biol Chem. 2014;50:29–40. PubMed PMID: 24569026. 24569026 10.1016/j.compbiolchem.2014.01.011

[5] Thykaer J, Andersen MR, Baker SE Essential pathway identification: from in silico analysis to potential antifungal targets in *Aspergillus fumigatus*. Med Mycol. 2009;47 Suppl 1:S80–S87. PubMed PMID: 19253142. 19253142 10.1080/13693780802455305

[6] Becker JM, Kauffman SJ, Hauser M, et al Pathway analysis of *Candida albicans* survival and virulence determinants in a murine infection model. Proc Natl Acad Sci U S A. 2010;107(51):22044–22049. PubMed PMID: 21135205; PubMed Central PMCID: PMCPMC3009777. 21135205 10.1073/pnas.1009845107 PMC3009777

[7] Ianiri G, Idnurm A Essential gene discovery in the basidiomycete *Cryptococcus neoformans* for antifungal drug target prioritization. MBio. 2015;6(2). PubMed PMID: 25827419; PubMed Central PMCID: PMCPMC4453551 DOI:10.1128/mBio.02334-14 PMC4453551 25827419

[8] Kaltdorf M, Srivastava M, Gupta SK, et al Systematic identification of anti-fungal drug targets by a metabolic network approach. Front Mol Biosci. 2016;3:22 PubMed PMID: 27379244; PubMed Central PMCID: PMCPMC4911368. 27379244 10.3389/fmolb.2016.00022 PMC4911368

[9] Sasse A, Hamer SN, Amich J, et al Mutant characterization and in vivo conditional repression identify aromatic amino acid biosynthesis to be essential for *Aspergillus fumigatus* virulence. Virulence. 2016;7(1):56–62. PubMed PMID: 26605426; PubMed Central PMCID: PMCPMC4871646. 26605426 10.1080/21505594.2015.1109766 PMC4871646

[10] Liebmann B, Muhleisen TW, Muller M, et al Deletion of the *Aspergillus fumigatus* lysine biosynthesis gene *lysF* encoding homoaconitase leads to attenuated virulence in a low-dose mouse infection model of invasive aspergillosis. Arch Microbiol. 2004;181(5):378–383. PubMed PMID: 15052376. 15052376 10.1007/s00203-004-0667-3

[11] Schobel F, Jacobsen ID, Brock M Evaluation of lysine biosynthesis as an antifungal drug target: biochemical characterization of *Aspergillus fumigatus* homocitrate synthase and virulence studies. Eukaryot Cell. 2010;9(6):878–893. PubMed PMID: 20363898; PubMed Central PMCID: PMCPMC2901645. 20363898 10.1128/EC.00020-10 PMC2901645

[12] Dietl AM, Amich J, Leal S, et al Histidine biosynthesis plays a crucial role in metal homeostasis and virulence of *Aspergillus fumigatus*. Virulence. 2016;7(4):465–476. PubMed PMID: 26854126; PubMed Central PMCID: PMCPMC4871644. 26854126 10.1080/21505594.2016.1146848 PMC4871644

[13] Amich J, Dumig M, O’Keeffe G, et al Exploration of sulfur assimilation of *Aspergillus fumigatus* reveals biosynthesis of sulfur-containing amino acids as a virulence determinant. Infect Immun. 2016;84(4):917–929. PubMed PMID: 26787716; PubMed Central PMCID: PMCPMC4807484. 26787716 10.1128/IAI.01124-15 PMC4807484

[14] Tao L, Gao N, Chen S, et al The *choC* gene encoding a putative phospholipid methyltransferase is essential for growth and development in *Aspergillus nidulans*. Curr Genet. 2010;56(3):283–296. PubMed PMID: 20379720. 20379720 10.1007/s00294-010-0300-8

[15] Fischer GJ, Keller NP Production of cross-kingdom oxylipins by pathogenic fungi: an update on their role in development and pathogenicity. J Microbiol. 2016;54(3): 254–264. PubMed PMID: 26920885; PubMed Central PMCID: PMCPMC5107414. 26920885 10.1007/s12275-016-5620-z PMC5107414

[16] Pontecorvo G, Roper JA, Hemmons LM, et al The genetics of *Aspergillus nidulans*. Adv Genet. 1953;5: 141–238. Epub 1953/01/01.PubMed PMID: 13040135. 13040135 10.1016/s0065-2660(08)60408-3

[17] Hartmann T, Dumig M, Jaber BM, et al Validation of a self-excising marker in the human pathogen *Aspergillus fumigatus* by employing the beta-rec/six site-specific recombination system. Appl Environ Microbiol. 2010;76(18):6313–6317. PubMed PMID: 20656854; PubMed Central PMCID: PMCPMC2937505. 20656854 10.1128/AEM.00882-10 PMC2937505

[18] Schrettl M, Beckmann N, Varga J, et al HapX-mediated adaption to iron starvation is crucial for virulence of *Aspergillus fumigatus*. PLoS Pathog. 2010;6(9):e1001124 Epub 2010/10/14. 20941352 10.1371/journal.ppat.1001124 PMC2947994

[19] Nierman WC, Pain A, Anderson MJ, et al Genomic sequence of the pathogenic and allergenic filamentous fungus *Aspergillus fumigatus*. Nature. 2005;438(7071):1151–1156. PubMed PMID: 16372009. 16372009 10.1038/nature04332

[20] Cerqueira GC, Arnaud MB, Inglis DO, et al The Aspergillus genome database: multispecies curation and incorporation of RNA-Seq data to improve structural gene annotations. Nucleic Acids Res. 2014;42(Database issue):D705–10. PubMed PMID: 24194595; PubMed Central PMCID: PMCPMC3965050. 24194595 10.1093/nar/gkt1029 PMC3965050

[21] Krappmann S, Sasse C, Braus GH Gene targeting in *Aspergillus fumigatus* by homologous recombination is facilitated in a nonhomologous end- joining-deficient genetic background. Eukaryot Cell. 2006;5(1):212–215. Epub 2006/01/10. 16400185 10.1128/EC.5.1.212-215.2006 PMC1360265

[22] Hartmann T, Dumig M, Jaber BM, et al Validation of a self-excising marker in the human pathogen *Aspergillus fumigatus* by employing the beta-rec/six site-specific recombination system. Appl Environ Microbiol. 2010;76(18):6313–6317. Epub 2010/07/27. 20656854 10.1128/AEM.00882-10 PMC2937505

[23] Garfoot AL, Zemska O, Rappleye CA *Histoplasma capsulatum* depends on de novo vitamin biosynthesis for intraphagosomal proliferation. Infect Immun. 2014;82(1):393–404. PubMed PMID: 24191299; PubMed Central PMCID: PMCPMC3911860. 24191299 10.1128/IAI.00824-13 PMC3911860

[24] Bonamore A, Boffi A Flavohemoglobin: structure and reactivity. IUBMB Life. 2008;60(1):19–28. Epub 2008/ 04/02. 18379989 10.1002/iub.9

[25] Schinko T, Berger H, Lee W, et al Transcriptome analysis of nitrate assimilation in *Aspergillus nidulans* reveals connections to nitric oxide metabolism. Mol Microbiol. 2010;78(3):720–738. PubMed PMID: 20969648; PubMed Central PMCID: PMCPMC3020322. 20969648 10.1111/j.1365-2958.2010.07363.x PMC3020322

[26] Haas H Iron - a key nexus in the virulence of *Aspergillus fumigatus*. Front Microbiol. Epub 2012/02/22 2012;3:28 PubMed PMID: 22347220; PubMed Central PMCID: PMC3272694. 22347220 10.3389/fmicb.2012.00028 PMC3272694

[27] Schrettl M, Bignell E, Kragl C, et al Distinct roles for intra- and extracellular siderophores during *Aspergillus fumigatus* infection. PLoS Pathog. 2007;3(9):1195–1207. PubMed PMID: ISI:000249768300003. 17845073 10.1371/journal.ppat.0030128 PMC1971116

[28] Eisendle M, Oberegger H, Zadra I, et al The siderophore system is essential for viability of *Aspergillus nidulans*: functional analysis of two genes encoding L-ornithine N-5-monooxygenase (sidA) and a non-ribosomal peptide synthetase (sidC). Mol Microbiol. 2003;49(2):359–375. PubMed PMID: ISI:000184224700007. 12828635 10.1046/j.1365-2958.2003.03586.x

[29] Oberegger H, Eisendle M, Schrettl M, et al 4 ‘-phosphopantetheinyl transferase-encoding npgA is essential for siderophore biosynthesis in *Aspergillus nidulans*. Curr Genet. 2003;44(4):211–215. PubMed PMID: ISI:000186823000005. 14508603 10.1007/s00294-003-0434-z

[30] Johns A, Scharf DH, Gsaller F, et al A nonredundant phosphopantetheinyl transferase, PptA, is a novel antifungal target that directs secondary metabolite, siderophore, and lysine biosynthesis in *Aspergillus fumigatus* and is critical for pathogenicity. MBio. 2017;8(4). PubMed PMID: 28720735; PubMed Central PMCID: PMCPMC5516258 DOI:10.1128/mBio.01504-16 PMC5516258 28720735

[31] Oberegger H, Eisendle M, Schrettl M, et al 4ʹ-phosphopantetheinyl transferase-encoding *npgA* is essential for siderophore biosynthesis in *Aspergillus nidulans*. Curr Genet. 2003;44(4):211–215. PubMed PMID: 14508603. 14508603 10.1007/s00294-003-0434-z

[32] Grahl N, Shepardson KM, Chung D, et al Hypoxia and fungal pathogenesis: to air or not to air? Eukaryot Cell. 2012;11(5):560–570. Epub 2012/ 03/27. 22447924 10.1128/EC.00031-12 PMC3346435

[33] Brown JS, Aufauvre-Brown A, Brown J, et al Signature-tagged and directed mutagenesis identify PABA synthetase as essential for *Aspergillus fumigatus* pathogenicity. Mol Microbiol. 2000;36(6): 1371–1380. PubMed PMID: 10931287. 10931287 10.1046/j.1365-2958.2000.01953.x

[34] Yekutiel A, Shalit I, Shadkchan Y, et al In vitro activity of caspofungin combined with sulfamethoxazole against clinical isolates of *Aspergillus* spp. Antimicrob Agents Chemother. 2004;48(9):3279–3283. PubMed PMID: 15328085; PubMed Central PMCID: PMCPMC514744. 15328085 10.1128/AAC.48.9.3279-3283.2004 PMC514744

[35] Bourne CR Utility of the biosynthetic folate pathway for targets in antimicrobial discovery. Antibiotics (Basel). 2014;3(1):1–28. PubMed PMID: 27025730; PubMed Central PMCID: PMCPMC4790348. 27025730 10.3390/antibiotics3010001 PMC4790348

[36] Balloy V, Huerre M, Latge JP, et al Differences in patterns of infection and inflammation for corticosteroid treatment and chemotherapy in experimental invasive pulmonary aspergillosis. Infect Immun. 2005;73(1):494–503. PubMed PMID: 15618189; PubMed Central PMCID: PMCPMC538925. 15618189 10.1128/IAI.73.1.494-503.2005 PMC538925

[37] Dick T, Manjunatha U, Kappes B, et al Vitamin B6 biosynthesis is essential for survival and virulence of *Mycobacterium tuberculosis*. Mol Microbiol. 2010;78(4):980–988. PubMed PMID: 20815826. 20815826 10.1111/j.1365-2958.2010.07381.x

[38] Reidling JC, Lambrecht N, Kassir M, et al Impaired intestinal vitamin B1 (thiamin) uptake in thiamin transporter-2-deficient mice. Gastroenterology. 2010;138(5):1802–1809. PubMed PMID: 19879271; PubMed Central PMCID: PMCPMC4916904. 19879271 10.1053/j.gastro.2009.10.042 PMC4916904

[39] Purnell DM The effects of specific auxotrophic mutations on the virulence of *Aspergillus nidulans* for mice. Mycopathol Mycol Appl. 1973;50(3): 195–203. PubMed PMID: 4580921. 4580921 10.1007/BF02053368

[40] Gudipati V, Koch K, Lienhart WD, et al The flavoproteome of the yeast *Saccharomyces cerevisiae*. Biochim Biophys Acta. 2014;1844(3):535–544. PubMed PMID: 24373875; PubMed Central PMCID: PMCPMC3991850. 24373875 10.1016/j.bbapap.2013.12.015 PMC3991850

[41] Lapp K, Vodisch M, Kroll K, et al Characterization of the *Aspergillus fumigatus* detoxification systems for reactive nitrogen intermediates and their impact on virulence. Front Microbiol. 2014;5:469 PubMed PMID: 25309516; PubMed Central PMCID: PMCPMC4160965. 25309516 10.3389/fmicb.2014.00469 PMC4160965

[42] Haas H Fungal siderophore metabolism with a focus on *Aspergillus fumigatus*. Nat Prod Rep. 2014;31(10):1266–1276. PubMed PMID: 25140791; PubMed Central PMCID: PMCPMC4162504. 25140791 10.1039/c4np00071d PMC4162504

[43] Leonardi R, Zhang YM, Rock CO, et al Coenzyme A: back in action. Prog Lipid Res. 2005;44(2–3):125–153. PubMed PMID: 15893380. 15893380 10.1016/j.plipres.2005.04.001

[44] Long Q, Ji L, Wang H, et al Riboflavin biosynthetic and regulatory factors as potential novel anti-infective drug targets. Chem Biol Drug Des. 2010;75(4):339–347. PubMed PMID: 20148904. 20148904 10.1111/j.1747-0285.2010.00946.x

[45] Chen J, Illarionov B, Bacher A, et al A high-throughput screen utilizing the fluorescence of riboflavin for identification of lumazine synthase inhibitors. Anal Biochem. 2005;338(1):124–130. PubMed PMID: 15707942. 15707942 10.1016/j.ab.2004.11.033

[46] Zhao Y, Bacher A, Illarionov B, et al Discovery and development of the covalent hydrates of trifluoromethylated pyrazoles as riboflavin synthase inhibitors with antibiotic activity against *Mycobacterium tuberculosis*. J Org Chem. 2009;74(15):5297–5303. PubMed PMID: 19545132. 19545132 10.1021/jo900768c

[47] Spry C, Kirk K, Saliba KJ Coenzyme A biosynthesis: an antimicrobial drug target. FEMS Microbiol Rev. 2008;32(1):56–106. PubMed PMID: 18173393. 18173393 10.1111/j.1574-6976.2007.00093.x

[48] Sambandamurthy VK, Wang X, Chen B, et al A pantothenate auxotroph of *Mycobacterium tuberculosis* is highly attenuated and protects mice against tuberculosis. Nat Med. 2002;8(10):1171–1174. PubMed PMID: 12219086. 12219086 10.1038/nm765

[49] Hung AW, Silvestre HL, Wen S, et al Optimization of inhibitors of *Mycobacterium tuberculosis* pantothenate synthetase based on group efficiency analysis. ChemMedChem. 2016;11(1):38–42. PubMed PMID: 26486566; PubMed Central PMCID: PMCPMC4949533. 26486566 10.1002/cmdc.201500414 PMC4949533

[50] Calder RB, Williams RS, Ramaswamy G, et al Cloning and characterization of a eukaryotic pantothenate kinase gene (*panK*) from *Aspergillus nidulan*s. J Biol Chem. 1999;274(4):2014–2020. PubMed PMID: 9890959. 9890959 10.1074/jbc.274.4.2014

[51] Ben Yaakov D, Shadkchan Y, Albert N, et al The quinoline bromoquinol exhibits broad-spectrum antifungal activity and induces oxidative stress and apoptosis in *Aspergillus fumigatus*. J Antimicrob Chemother. 2017;72(8):2263–2272. PubMed PMID: 28475687. 28475687 10.1093/jac/dkx117

