# Supplementary material for: Riboflavin and pantothenic acid biosynthesis are crucial for iron homeostasis and virulence in the pathogenic mold Aspergillus fumigatus
Source: Virulence. 2018 Jul 27;9(1):1036–49. doi: 10.1080/21505594.2018.1482181 (PMC6068542; doi:10.1080/21505594.2018.1482181)
Supplement: Supplemental Material [file kvir-09-01-1482181-s001.docx]

**Supplementary**

**Generation of the *A. fumigatus* mutant strains and their verification by Southern analysis**. The bipartite marker strategy was used for *riboB* and *panA* gene deletion and reconstitution strains. Therefore, the coding sequence of *riboB* (AFUA_1G13300) and *panA* (AFUA_5G11040) respectively, was replaced by a hygromycin (*hph*) resistance cassette in AfS77. Deletion constructs were made by joining three PCR fragments: 5´flank (using primers RiboB-5f-Fwd and RiboB-5f-Rev, PanA-5f-Fwd and PanA-5f-Rev), 3´flank (using primers RiboB-3f-Fwd and RiboB-3f-Rev, PanA-3f-Fwd and PanA-3f-Rev) and a hygromycin cassette, amplified from plasmid pAN7.1 (Punt, et al. 1987) using primers RiboB-hph-Fwd and RiboB-hph-Rev, PanA-hph-Fwd and PanA-hph-Rev). For this process the GeneArt® Seamless PLUS Cloning and Assembly Kit (Thermo Fisher) was applied. For the generation of each deletion strain, the three DNA inserts were recombined into a linear pUC19L (Thermo Fisher) vector and the two transformation fragments were amplified from the ligation product using primers RiboB-5f-Fwd and hph14-Rev, PanA-5f-Fwd and hph14-Rev for the 5´flank and RiboB-3f-Rev and hph15-Fwd, PanA-3f-Rev and hph15-Fwd for the 3´flank. For reconstitution of Δ*riboB* and Δ*panA*, a PCR fragment containing a complete *riboB* and *panA* copy, respectively, was amplified from genomic DNA using primers RiboB-5f-Fwd and Ribob-3f-Rev, PanA-3f-Fwd and PanA-3f-Rev. These PCR fragments were subcloned into the pCR2.1-TOPO^TM^TOPO vector (Thermo Fisher). The resulting *priboB* plasmid was linearized with *BsrGI* and the *ppanA* plasmids was linearized with *BlpI*, respectively to foster homologous recombination in the 3´-riboB and 3´-panA flanking region, respectively. The linearized plasmids were introduced into the Δ*riboB* and Δ*panA* strains, respectively, by protoplast transformation with selection for riboflavin and pantothenic acid, respectively. All primer used for cloning are listed in Supplementary Table 1.

The Gibson assembly strategy was used for *pyroA* and *thiB* gene deletion and reconstitution strains in *A. fumigatus*. Deletion constructs were made by joining four PCR fragments: 5' flank (using primers pyroA-5f-Fwd and pyroA-5f-Rev, ThiB-5f-Fwd and ThiB-5f-Rev), 3' flank, (using primers pyroA-3f-Fwd and pyroA-3f-Rev, ThiB-3f-Fwd and ThiB-3f-Rev), hygromycin cassette (using primers HygB_Fwd and HygB_Rev) and pUC19 (using primers pUC19L-Fwd and pUC19L-Rev) Vector (Supplementary Table 1). A unique *Asc*I restriction site, introduced into primers pyroA-5f-Fwd, pyroA-3f-Rev, ThiB-5f-Fwd and ThiB-3f-Rev, was later used to linearize the construct for transformation. The plasmids used for gene reconstitution were prepared by PCR amplification of *pyroA* and *thiB* coding and flanking regions using primers pyroA-KI-Fwd, pyroA-KI-Rev (*pyroA*) and ThiB-KI-Fwd, ThiB-KI-Rev (*thiB*) and ligation into the pGEM-T vector. These plasmids were used to complement the *ΔpyroA* and *ΔthiB* strains under pyridoxine and thiamine selection, respectively.

*A. fumigatus* transformation was performed according to Tilburn et al. (Tilburn, et al. 1983). A hygromycin B concentration of 0.1 mg∙ml^-1^ (Calbiochem) was applied for selection on minimal medium plates. Transformants were pre-analyzed based on vitamin auxotrophy and confirmed by Southern blot analysis or PCR (Supplementary Fig. 2A-M).

**Supplementary Fig. 1. Key vitamin B biosynthetic pathways in *A. fumigatus***. Putative schematic outline of the riboflavin (A), pantothenic acid (B), pyridoxine (C) and thiamine (D) biosynthetic pathways in *A. fumigatus*. Enzymatic activities are shown in black boxes. The *A. fumigatus* proteins comprising the respective enzymatic activities are framed in black. The genes investigated in the current study, are shaded in yellow

1. Riboflavin biosynthetic pathway


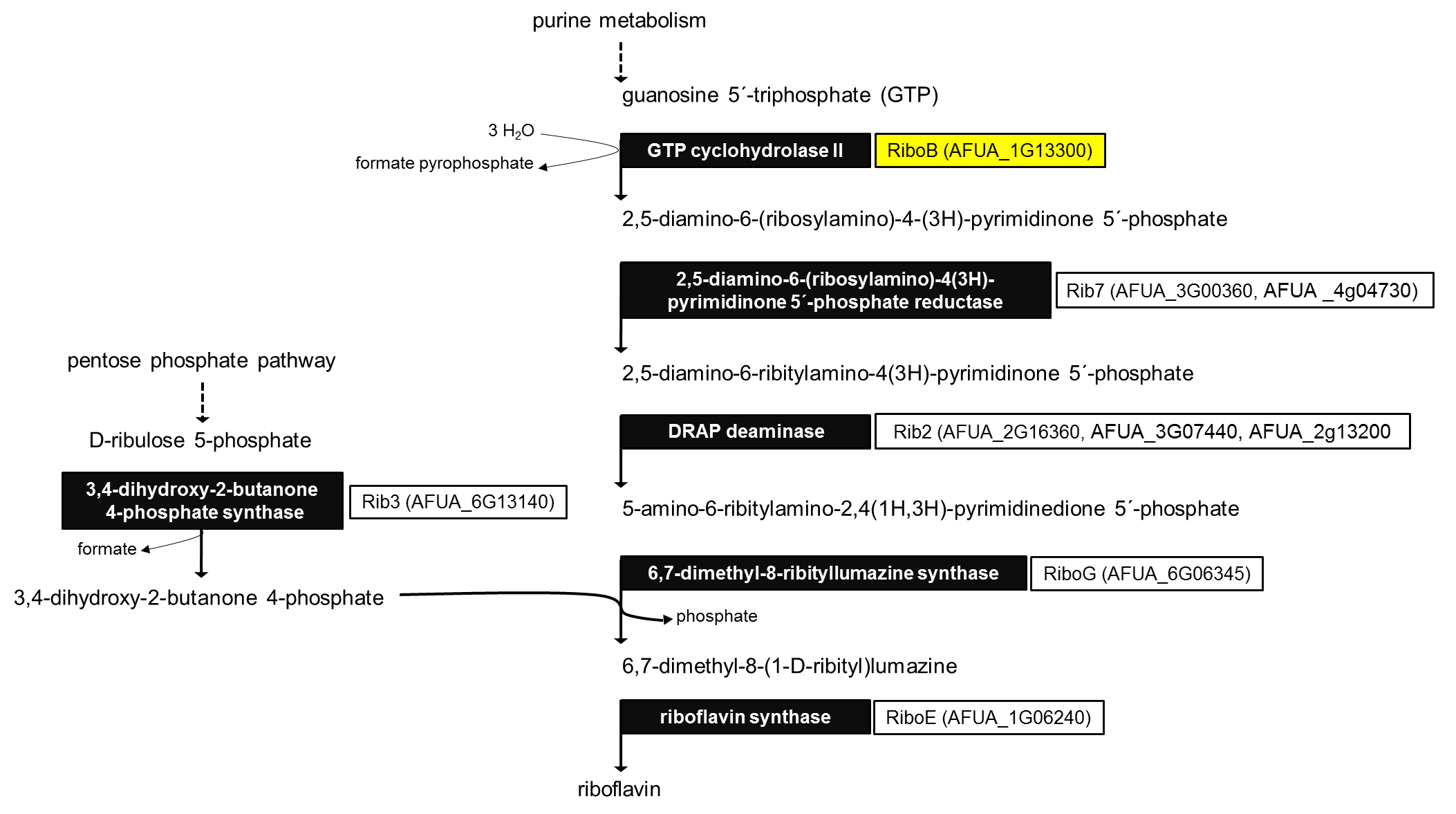


1. Pantothenic acid biosynthetic pathway


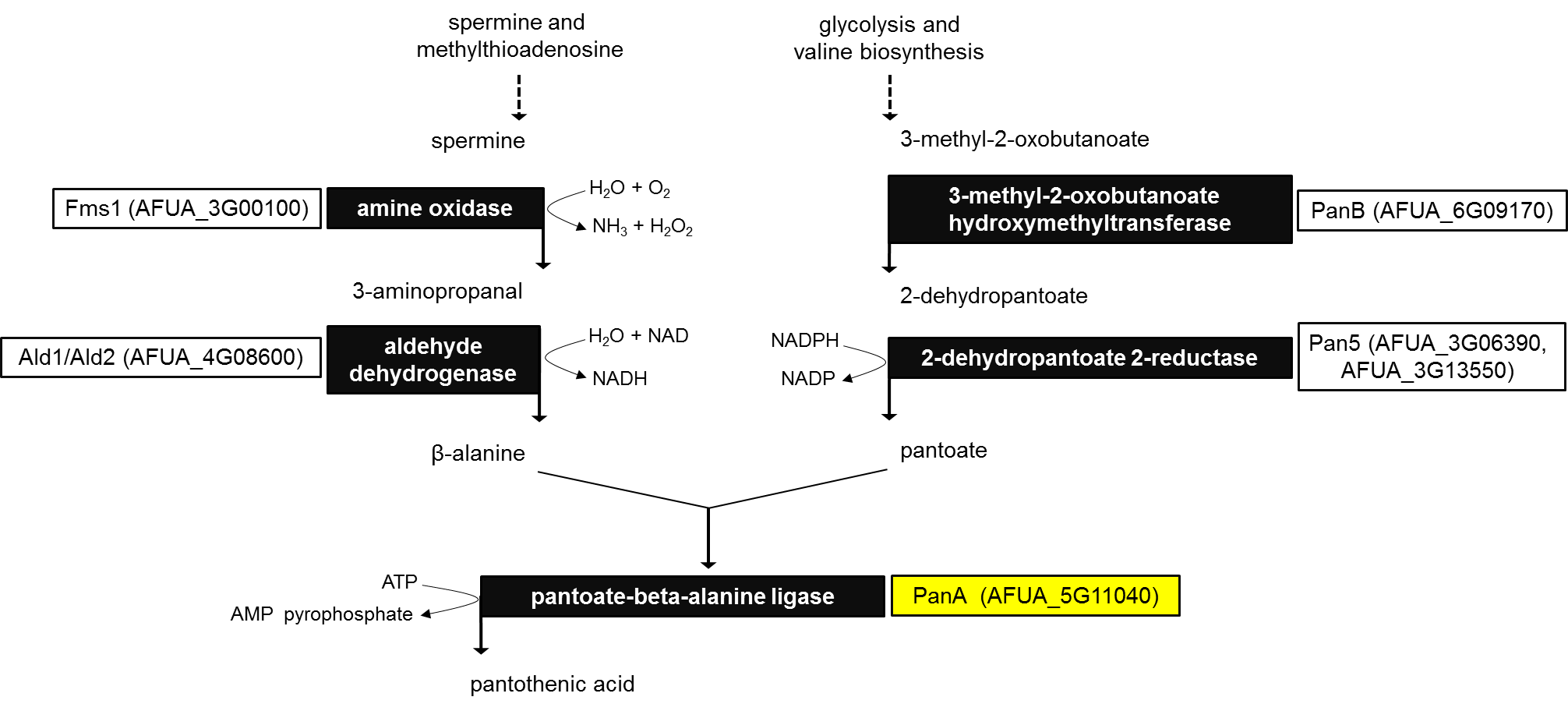


1. Pyridoxine biosynthetic pathway


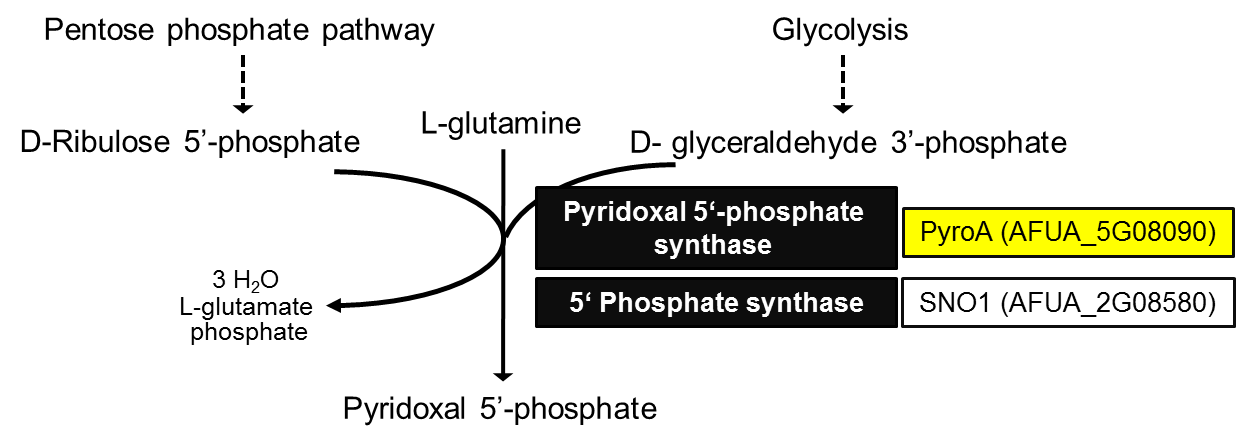


1. Thiamine biosynthetic pathway


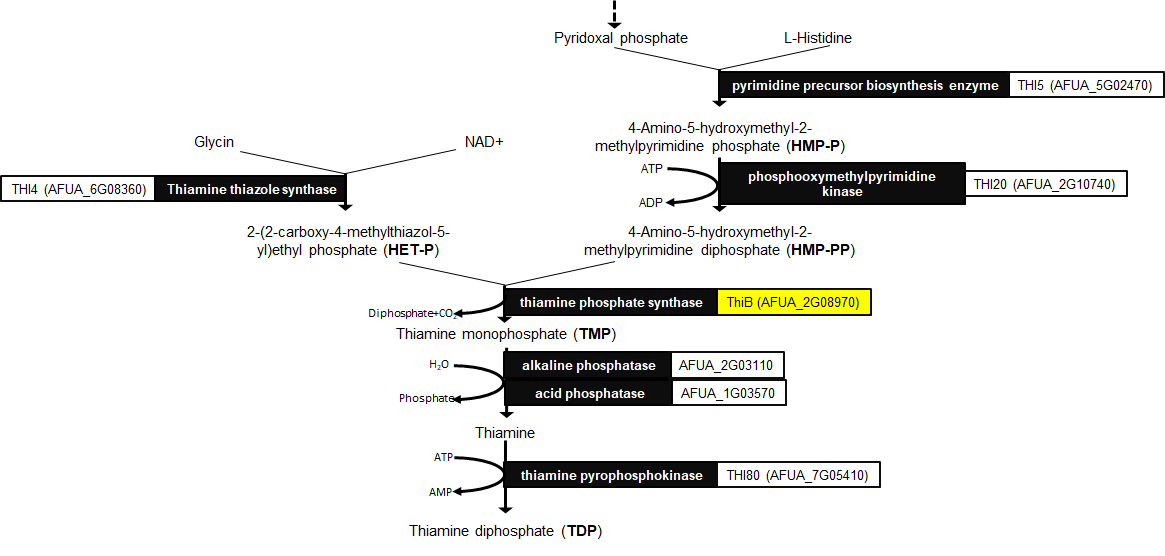


**Supplementary Fig. 2. Deletion and reconstitution of the genes described in this study**. Deletion of *riboB* (A-C) and *panA* (D-F) was performed using the split marker technique. Deletion of *pyroA* (G-I) and *thiB* (J-M) was performed using the conventional single marker method. Complementation was performed as detailed in the Materials and Methods. Southern analysis was performed to verify gene deletion and complementation.


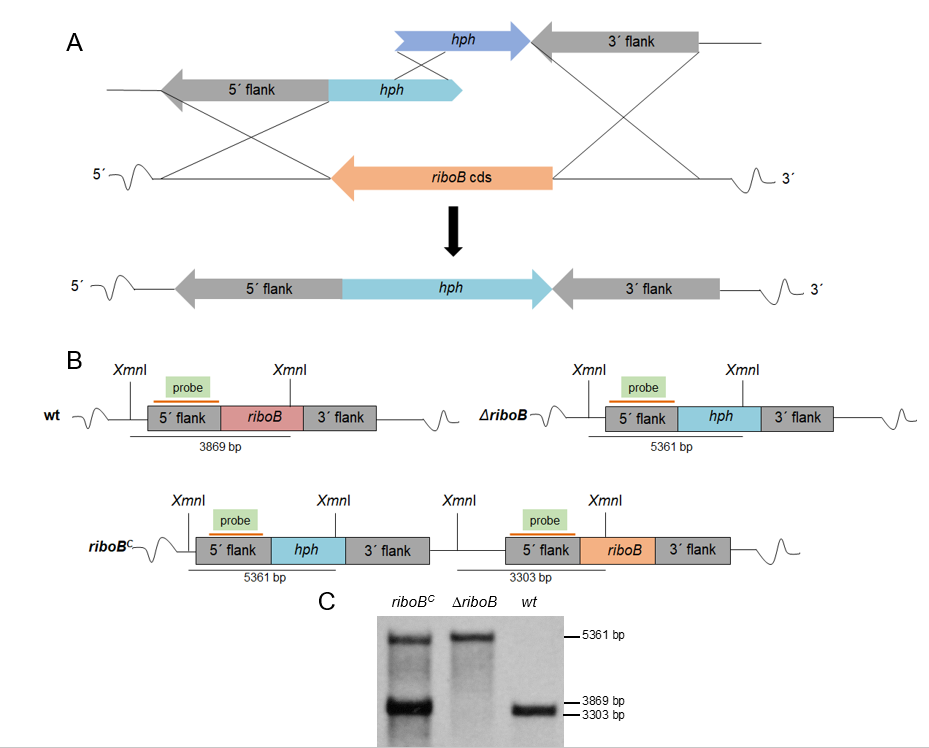


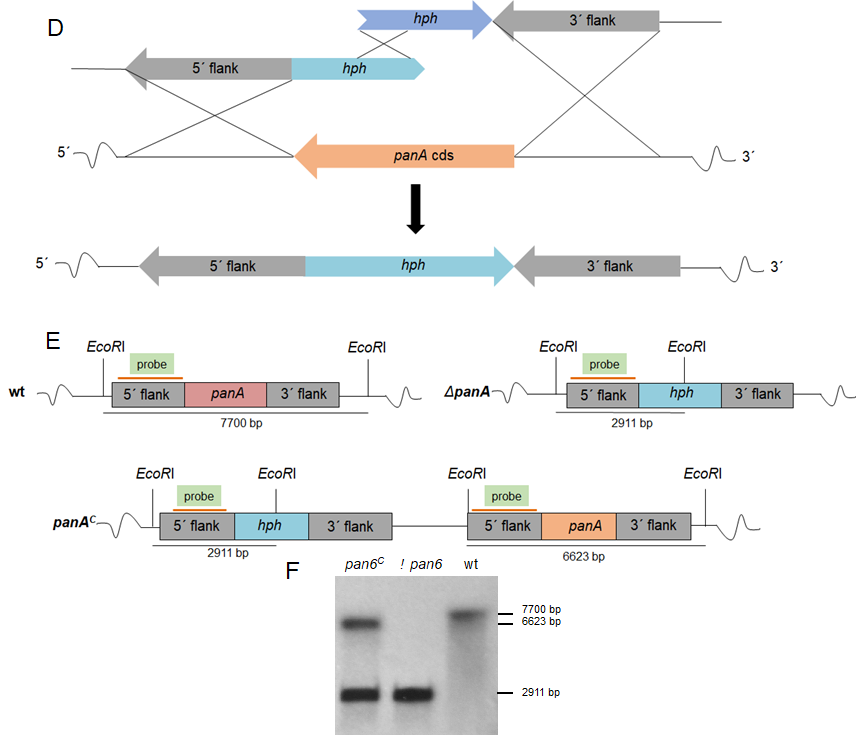


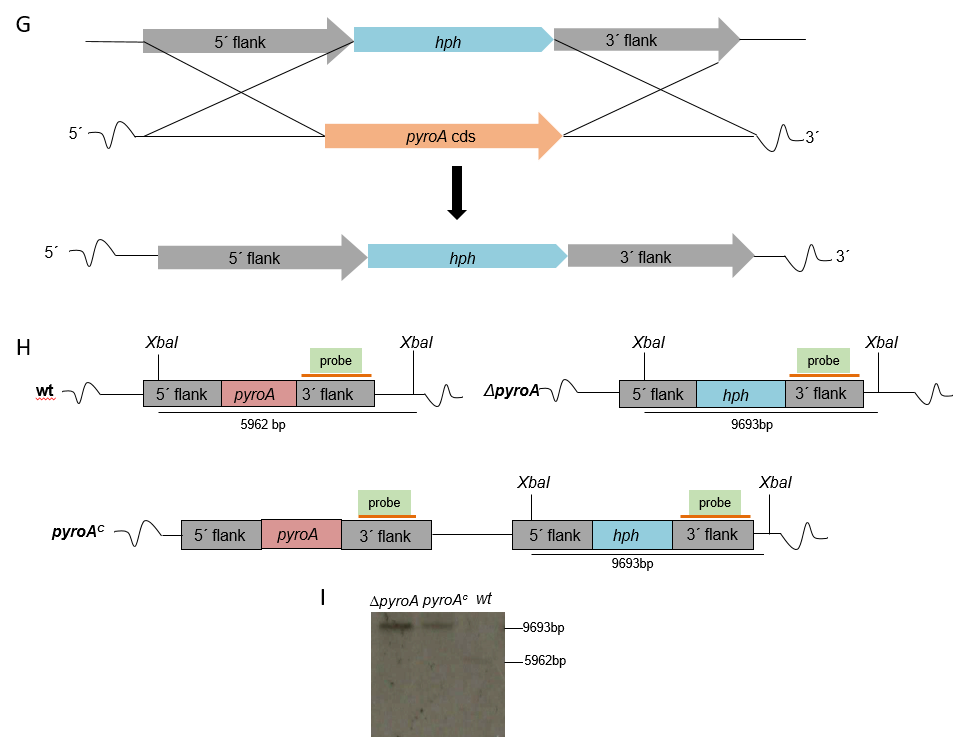


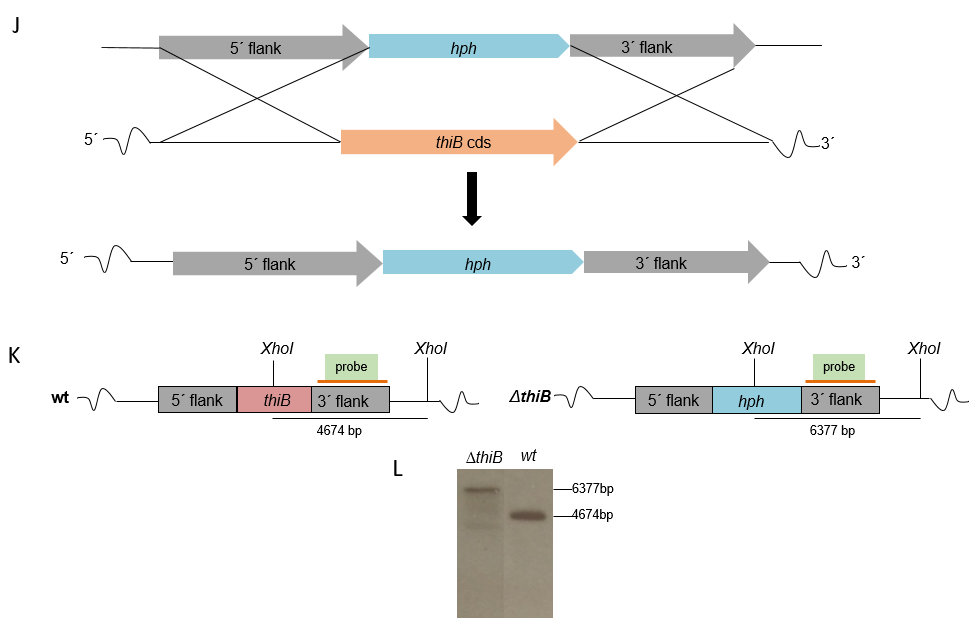


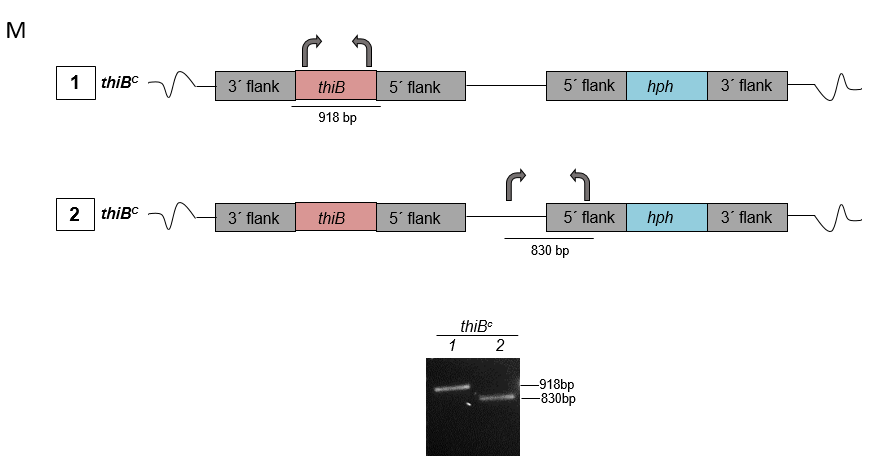


**Supplementary Fig. 3. Conidial viability and germination of *ΔriboB* grown on MM supplemented with 2.5 µM riboflavin, remains the same as wt.** *ΔriboB* and wt (AfS77) strains were cultured on MM supplemented with 2.5 µM riboflavin for 48 h at 37˚C. Spores were collected and germinated in either MM without vitamins (MM), MM + 2.5 µM riboflavin, or MM + vitamins (2.5 µM pantothenic acid, 10 µM pyridoxine, 10 µM niacin and 2.5 µM riboflavin). Vitamin or riboflavin supplementation resulted in normal germination, as characterized by the fraction of germinating spores and the extent of hyphal growth.


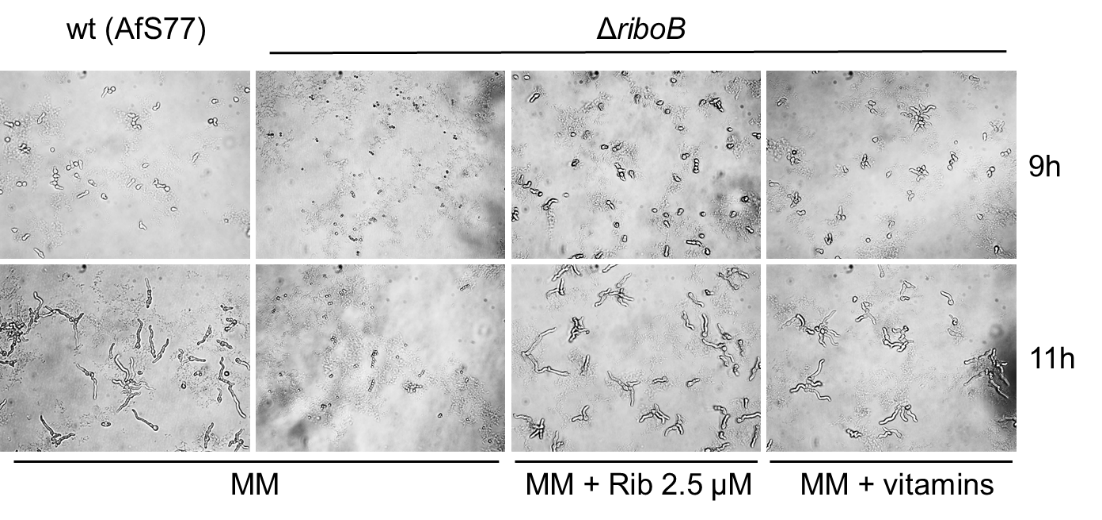


Supplementary Table 1. Primers used in this study.

| **Primer** | **Sequence 5´-3** |
| --- | --- |
| RiboB-5f-Fwd | AATTCGAGCTCGGTACGGGGAGGGGATCCAG |
| RiboB-5f-Rev | TACCTAGGTGCGATGACGGACTT |
| RiboB-hph-Fwd | TCATCGCACCTAGGTACAGAAGTCC |
| RiboB-hph-Rev | GGATAGGTTCTAGAAAGAAGGATTACCT |
| RiboB-3f-Fwd | TTTCTAGAACCTATCCCCTTTCGG |
| RiboB-3f-Rev | GCCAAGCTTGCATGCCTTGGGGGATGGTGGT |
| hph15-Rev | GAGAGCCTGACCTATTGC |
| hph14-Fwd | TCTCGTCTTCCTCATTCTC |
| PanA-5f-Fwd | AATTCGAGCTCGGTACTCCGATCACATCGTTG |
| PanA-5f-Rev | TACCTAGGTGTGGAATTGAACAACG |
| PanA-hph-Fwd | ATTCCACACCTAGGTACAGAAGTCC |
| PanA-hph-Rev | GTGAATGGTCTAGAAAGAAGGATTACCT |
| PanA-3f-Fwd | TTTCTAGACCATTCACAAACATCAATC |
| PanA-3f-Rev | GCCAAGCTTGCATGCCCTTCAGCTCCAGCTTC |
| PyroA-3f-Fwd | TAATAAAACCCATCTAGTGCTTGCACTACTATCTCTACTTGTG |
| PyroA-3f-Rev | TTACGCCAAGCTTGCATGCCGGCGCGCCACGATCCTTCTCTCCATCC |
| PyroA-5f-Fwd | AGTGAATTCGAGCTCGGTACGGCGCGCCTTGGAAGCGGTCAGGATA |
| PyroA-5f-Rev | CTATAAAACCTGAGTGATGCTCTGTGTATTTAGAAGGGAATGG |
| ThiB-5f-Fwd | AGTGAATTCGAGCTCGGTACGGCGCGCCGGAGAGTTGGGATCGTTTCA |
| ThiB-5f-Rev | CTATAGGACCTGAGTGATGCTTTCTATTGCCATTGTCAGCAT |
| ThiB-3f-Fwd | TAATATGGTCCATCTAGTGCTCCATAGTGTATATAGACGATGTGT |
| ThiB-3f-Rev | TTACGCCAAGCTTGCATGCCGGCGCGCCGACGGTCCGTGATGTAGAAA |
| HygB_Fwd | GCATCACTCAGGTCCTATAGGTCAA |
| HygB_Rev | GCACTAGATGGACCATATTATGCTCAACT |
| pUC19L-Fwd | GGCATGCAAGCTTGGCGTAA |
| pUC19L-Rev | AGTGAATTCGAGCTCGGTAC |
| pyroA-KI-Fwd | ATGGGTTGGGAGTCAGGAT |
| pyroA-KI-Rev | ATCATTACGCGGAGATGTGAAG |
| ThiB-KI-Fwd | AGCCAAGACTCGCTGAGAAA |
| ThiB-KI-Rev | AGAGATGGGAGGCGTACAATAAG |

**Supplementary Table 2. Strains of *A. fumigatus* used in this study**.

| Strain | Genotype |
| --- | --- |
| AfS77 (wt) | Wild-type; *akuA::loxP* derived from ATCC 46645 |
| *∆riboB* | *Afu1g13300::hph* |
| *riboB^C^* | *Afu1g13300::hph;Afu1g13300* |
| *∆panA* | *Afu5g11040::hph* |
| *panA^C^* | *Afu5g11040::hph;Afu5g11040* |
| *∆pyroA* | *Afu5g08090::hph* |
| *pyroA^C^* | *Afu5g08090::hph;Afu5g08090* |
| *∆thiB* | *Afu2g08970::hph* |
| *thiB^C^* | *Afu2g08970::hph; Afu2g08970* |

**Supplementary Table 3.** Riboflavin biosynthetic genes in *A. fumigatus* and human homologs identified by blastp searches.

| **Enzyme name** | **Gene^1^** | ***A. fumigatus homolog*** | ***Homo sapiens* homolog** | **Blastp  E-value** | **% identity** |
| --- | --- | --- | --- | --- | --- |
| GTP cyclohydrolase II^2^ | *rib1/riboB* | AFUA _1G13300 | - | no similarity | 0 |
| 2,5-diamino-6-(ribosylamino)-4(3H)-pyrimidinone 5'-phosphate reductase | *rib7* | AFUA _3G00360 | - | no similarity | 0 |
|  |  | AFUA _4G04730 | ACA00811.1 | 4.3 | 34% (15/44  of 622 aa) |
| 2,5-diamino-6-(ribitylamino)-4(3H)-pyrimidinone 5'-phosphate reductase (DRAP) deaminase^3^ | *rib2* | AFUA _2G16360 | BAG63178.1 | 2e-79 | 35%  (157/451 of 524 aa) |
|  |  | AFUA_3G07440 | NP_078824.2 | 3.0 | 56%  (14/25 of 865 aa) |
|  |  | AFUA_2G13200 |  | no similarity |  |
| 6,7-dimetyhl-8-ribityllumazine synthase | *rib4/riboG* | AFUA _6G06345 | - | no similarity | 0 |
| Riboflavin synthase | *rib5/riboE* | AFUA _ 1G06240 | - | no similarity | 0 |
| 3,4-dihydroxy-2-butanone 4-phosphate synthase | *rib3* | AFUA _6G13140 | - | no similarity | 0 |

^1^ *S. cerevisiae/A. nidulans* nomenclature

^2^ deletion in *A. fumigatus* resulted in riboflavin auxotrophy

^3^ deletion of AFUA _2g16360 did not result in riboflavin auxotrophy, possibly due to the presence of paralogs

**Supplementary Table 4.** Pantothenic acid biosynthetic genes in *A. fumigatus* and human homologs identified by blastp searches.

| **Enzyme name** | **Gene^1^** | ***A. fumigatus homolog*** | ***Homo sapiens* homolog** | **blastp  E-value** | **% identity** |
| --- | --- | --- | --- | --- | --- |
| 3-methyl-2-oxobutanoate hydroxymethyltransferase^2^ | *pan2/panB* | AFUA _6G09170 | - | no similarity | 0 |
| 2-dehydropantoate 2-reductase | *pan5* | AFUA _3G06390 | - | no similarity | 0 |
|  |  | AFUA _3G13550 | - | no similarity | 0 |
| Pantoate-beta-alanine ligase | *pan6*/panA | AFUA_5G11040**^2^**  *panA* | - | no similarity | 0 |
| Amine oxidase | *fms1* | AFUA_3G00100**^3^** | 2Z5XA | 9e-30 | 26% (98/380 of 513 aa) |
| Aldehyde dehydrogenase | *ald2/ald3* | AFUA_4G08600**^4^** | NP_000680.2 | 3e-180 | 51% (260/508 of 550 aa) |

^1^ *S. cerevisiae/A. nidulans* nomenclature

^2^ Deletion in *A. fumigatus* resulted in pantothenic acid auxotrophy

^3^ *A. fumigatus* comprises numerous homologs of *fms1*

^4^ *A. fumigatus* comprises numerous homologs of *ald2/ald3*

**Supplementary Table 5.** Pyridoxine biosynthetic genes in *A. fumigatus* and human homologs identified by blastp searches.

| **Enzyme name** | **Gene^1^** | ***A. fumigatus homolog*** | ***Homo sapiens* homolog** | **blastp  E-value** | **identity** |
| --- | --- | --- | --- | --- | --- |
| Pyridoxal 5‘-phosphate synthase | *Snz3/*  *pyroA* | AFUA_5G08090/  *pyroA* | - | no similarity | 0 |
| 5‘ Phosphate synthase | Sno1 | AFUA_2G08580 | - | no similarity | 0 |

^1^ *S. cerevisiae/A. nidulans* nomenclature

**Supplementary** **Table 6.** Thiamine biosynthetic genes in *A. fumigatus* and human homologs identified by blastp searches.

| **Enzyme name** | **gene^1^** | ***A. fumigatus homolog*** | ***Homo sapiens* homolog** | **Blastp  E-value** | **% identity** |
| --- | --- | --- | --- | --- | --- |
| Pyrimidine precursor biosynthesis enzyme | *thi5* | AFUA_5G02470 | - | no similarity | 0 |
| Phosphooxymethylpyrimidine kinase | *thi20* | AFUA_2G10740 | - | no similarity | 0 |
| Thiamine phosphate synthase^2^ | *thi6/thiB* | AFUA_2G08970/  *thiB* | - | no similarity | 0 |
| Thiamine thiazole synthase | *thi4* | AFUA_6G08360 | BAG65581.1 | 7.3 | 34% (17/50  of 332 aa) |
| Alkaline phosphatase |  | AFUA_2G03110 | AAH14139.1 | 3e-27 | 31% (106/338 of 607 aa) |
| Acid phosphatase |  | AFUA_1G03570 | - | no similarity | 0 |
| Thiamine pyro  phosphokinase | *thi80* | AFUA_7G05410 | AAH68460.1 | 6e-37 | 33% (86/257  of 329 aa) |

^1^ *S. cerevisiae/A. nidulans* nomenclature

**References**

Punt, P. J., et al. 1987 Transformation of Aspergillus based on the hygromycin B resistance marker from *Escherichia coli*. Gene 56(1):117-24.

Tilburn, J., et al. 1983 Transformation by integration in *Aspergillus nidulans*. Gene 26(2-3):205-21.
